# Identification of a myotropic AAV by massively parallel in vivo evaluation of barcoded capsid variants

**DOI:** 10.1038/s41467-020-19230-w

**Published:** 2020-10-28

**Authors:** Jonas Weinmann, Sabrina Weis, Josefine Sippel, Warut Tulalamba, Anca Remes, Jihad El Andari, Anne-Kathrin Herrmann, Quang H. Pham, Christopher Borowski, Susanne Hille, Tanja Schönberger, Norbert Frey, Martin Lenter, Thierry VandenDriessche, Oliver J. Müller, Marinee K. Chuah, Thorsten Lamla, Dirk Grimm

**Affiliations:** 1 Heidelberg University Hospital, Dept. of Infectious Diseases/Virology, Cluster of Excellence CellNetworks, 69120 Heidelberg, Germany; 2 BioQuant, University of Heidelberg, 69120 Heidelberg, Germany; 3 Vrije Universiteit Brussel, Department of Gene Therapy & Regenerative Medicine, 1090 Brussels, Belgium; 4 University Hospital Schleswig-Holstein, Campus Kiel, Innere Medizin III, 24105 Kiel, Germany; 5 German Center for Infection Research (DZIF) and German Center for Cardiovascular Research (DZHK), partner site Hamburg/Kiel/Lübeck, 24105 Kiel, Germany; 6 Boehringer Ingelheim Pharma GmbH & Co. KG, Drug Discovery Sciences, 88400 Biberach an der Riß, Germany; 7 University of Leuven, Center for Molecular & Vascular Biology, Department of Cardiovascular Sciences, Leuven, 3000 Belgium; 8 German Center for Infection Research (DZIF) and German Center for Cardiovascular Research (DZHK), partner site Heidelberg, 69120 Heidelberg, Germany

**Keywords:** Gene delivery, Gene therapy

## Abstract

Adeno-associated virus (AAV) forms the basis for several commercial gene therapy products and for countless gene transfer vectors derived from natural or synthetic viral isolates that are under intense preclinical evaluation. Here, we report a versatile pipeline that enables the direct side-by-side comparison of pre-selected AAV capsids in high-throughput and in the same animal, by combining DNA/RNA barcoding with multiplexed next-generation sequencing. For validation, we create three independent libraries comprising 183 different AAV variants including widely used benchmarks and screened them in all major tissues in adult mice. Thereby, we discover a peptide-displaying AAV9 mutant called AAVMYO that exhibits superior efficiency and specificity in the musculature including skeletal muscle, heart and diaphragm following peripheral delivery, and that holds great potential for muscle gene therapy. Our comprehensive methodology is compatible with any capsids, targets and species, and will thus facilitate and accelerate the stratification of optimal AAV vectors for human gene therapy.

## Abstract (web summary)

Adeno-associated virus is the basis of many gene therapies and gene transfer vectors. Here the authors report a pipeline to enable side-by-side comparison of pre-selected capsids in a high throughput manner.

## Introduction

From a clinical perspective, an ideal AAV vector should specifically and efficiently express high levels of the therapeutic transgene product in the desired target tissue, following a single peripheral delivery of low particle doses. This will alleviate demands on vector manufacturing and concurrently improve patient safety and compliance. To obtain such superior vectors, others and we have previously devised and applied a variety of different technologies, permitting the molecular evolution and selection of designer AAVs^1^. Typically, this is accomplished by creating comprehensive capsid libraries for subsequent screening under positive and/or negative selection pressure. Despite the undisputed potential of this approach, a common observation is that even the most stringent primary selection schemes typically yield a diverse collection of interesting candidates rather than a single lead, which complicates the selection of (an) optimal candidate(s) for further (pre-)clinical development. In turn, this invariably creates a need for downstream and more focused, secondary procedures to narrow down the best capsid(s) in a robust, reliable, sensitive, and high-throughput fashion. Ideally, this is achieved by performing a secondary screen directly in animals and in a multiplexed manner, to facilitate head-to-head comparisons of in vivo tissue or cell specificities of different AAV capsids. Concurrently, such a simultaneous analysis in the same animal will substantially reduce the number of animals that are typically required for in vivo screens. Moreover, this methodology should enable a direct comparison of the capsids’ ability to deliver vector DNA to their expression of the encoded mRNA in the target cells, allowing for the rapid identification of the clinically most relevant candidates. Finally, the technology should include an assortment of wild type, naturally occurring AAV isolates, and published AAV variants, to enable assessment of relative improvements over established benchmarks in an all-inclusive manner. Thereby, it permits to conclusively stratify pre-selected AAV capsids under physiological and identical conditions, and it will thus help to realize the full potential of directed AAV capsid evolution.

Here, we introduce a combined experimental and bioinformatic workflow that fulfills all these requirements, and showcase its potential through the discovery of a synthetic AAV capsid termed AAVMYO that holds significant potential for gene transfer into the musculature, including skeletal muscle, heart, and diaphragm following peripheral delivery.

## Results

**Fig. 1:**
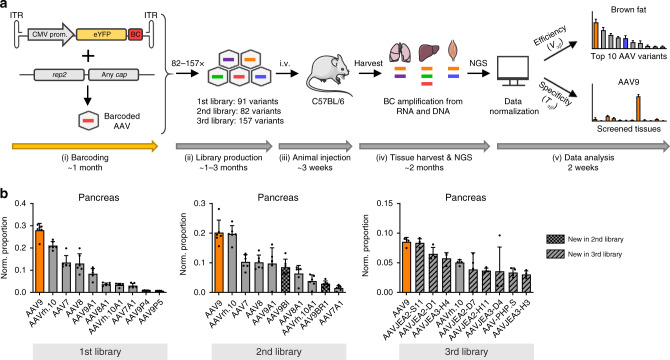
A robust workflow for massively parallel in vivo AAV capsid stratification. **a** Experimental setup. Shown are the essential experimental and bioinformatic steps, from the (i) cloning of barcoded YFP reporter vectors, (ii) library production, and (iii) animal (mouse here) injection, to (iv) tissue harvest and NGS, followed by (v) data analysis. Also shown is a timeline for the entire workflow or the individual steps. Note that the exact time required in step (ii) depends on the size of the library. Likewise, the time to perform the final bioinformatic analysis in step (v) is determined by available computing (IT) power. The first step (orange arrow) can be skipped if users start with our pre-existing collection of barcoded vector genomes, which will cut down the required overall time to <6 months. Please see the main text for further details. **b** Ranking of capsids in all three libraries by transcriptional efficiency (*V*_*αβ*_) in the pancreas. Shown are the top ten AAV variants and the proportion of their transcriptional efficiency after normalization to all capsids or barcodes, respectively, in each library. Depicted values are the average from six C57BL/6 J mice with SD. BC, barcode; i.v., intravenous. This figure contains clipart from Servier Medical Art. Source data are available in the Source data file.

To address the need of selecting the best AAV capsids for in vivo gene delivery in focused, secondary screens and to thereby complement current primary screening approaches, we established the comprehensive, experimental, and bioinformatic pipeline illustrated in Fig. 1a. Its hallmark is that AAV vector genomes (vg) packaged into capsid variants of interest or well-known benchmarks are first barcoded, and then qualitatively and quantitatively tracked in transduced animals by next-generation sequencing (NGS) at both, the DNA and RNA level. To this end, we inserted 159 distinct barcodes into the 3′ untranslated region of a yellow fluorescent protein (YFP) reporter driven by the ubiquitously active cytomegalovirus (CMV) promoter and encoded in a self-complementary AAV genome. During vector production, each barcode was assigned to a unique AAV capsid from the list of 183 variants in Supplementary Table 1, comprising 12 AAV wild types (AAV1 to AAV9, AAVrh.10, AAVpo.1, AAV12), as well as 94 peptide display mutants and 71 chimeric capsids created through DNA family shuffling^2^. The synthetic capsids have previously been isolated by others or us in specific tissues (e.g., AAV-PHP.B^3^, AAV2-ESGHGYF^4^, AAVM41^5^, AAV-LK03^6^, AAV-DJ^7^, AAV2-BR1^8^, AAV_587_MTP^9^, AAV-Anc80L65^10^, AAV2-7m8^11^, AAV2HBKO^12^, AAV2YF^13^, or AAV6.2^14^) or have emerged in our recent screens of AAV libraries in cultured cells or in murine liver or muscle, respectively^15^. This includes a set of 12 AAV serotypes that we have previously modified by insertion of over 20 different peptides in exposed capsid loops, and that we have recently studied and characterized extensively in cultured cell lines or primary cells^15^. It was thus very interesting and relevant to now also assess the performance of the best of these synthetic capsids in vivo. Vice versa, together with the other variants in Supplementary Table 1, these candidates provided an optimal assortment of diverse AAV capsids to validate the power and potential of our entire pipeline.

Over the course of this work, all barcoded capsids were consecutively pooled in different combinations to yield three distinct libraries, encompassing 91, 82, or 157 variants in the first, second, or third library, respectively (Supplementary Fig. 1, and Supplementary Tables 1 and 2; note that some of the 159 barcodes were recycled between the libraries and assigned to distinct capsids).

Next to composition, the three libraries also differed in their manufacturing process. For the first library, each vector was produced in two 15 cm dishes, and all resulting cell lysates were pooled without individual titration and purified on a single cesium chloride density gradient. Subsequent quality control by NGS revealed up to 3600-fold differences in the abundance of a specific barcode for individual capsids versus the mean, most evident for peptide-modified AAV6 and AAV12, or capsids with the 9-mer insertion CDCRGDCFC (peptide P2 in Supplementary Fig. 1 and Supplementary Table 1, and as reported^15^). Considering that the 91 variants in this first library were not titrated prior to pooling and that AAV capsids are widely known to produce with different efficiencies (in particular, AAV6 is hard to scale-up), we were not surprised to observe this variation. A detailed discussion of possible mechanisms underlying the differences in individual titers is beyond our scope, but we note that reasons can include varying capsid (thermo-)stabilities, or distortion of capsid conformation upon peptide insertion or capsid protein shuffling. In our most recent work^15^, we have moreover identified a specific peptide (P6, not included in the in vivo screens reported here) that most likely triggered sequestration of P6-displaying capsids by intracellular structures in the producer cells, offering another interesting mechanism that can explain heterogeneous AAV titers.

Consequently, for the second and third libraries, the number of cell culture dishes was adjusted to ensure that each capsid was produced in amounts >1 × 10^11^ vg, based on our prior experience with the manufacturing of all AAV variants. Moreover, we excluded or replaced capsids that produced poorly, including the aforementioned examples. Finally, from the lessons learned with the first library, we now purified each vector individually by iodixanol density gradient centrifugation prior to pooling all in equal amounts for concentration and dialysis. As hoped for, NGS analysis of these two libraries showed a far more homogeneous distribution of the 82 or 157 variants with 6.4- or 7.4-fold deviation from the theoretical mean proportion, respectively (Supplementary Fig. 1). This remaining deviation from perfect homogeneity likely results from the combination of two factors, namely, the error margin of the quantitative (q)PCR used for titration of the input AAV stocks, plus it is conceivable that individual capsids slightly varied in their losses during purification. Either way, we trust a less than eightfold variation in final titers between >150 different, co-purified capsids in a single library to be reasonable.

Next, we established a comprehensive bioinformatics-based pipeline comprising a multistep normalization strategy to quantify two essential parameters for each capsid in the libraries on the DNA and mRNA level: (i) the efficiency of functional transduction within a single tissue, and (ii) the specificity of transduction across all studied tissues. As explained in detail in the Supplementary Discussion, our normalization pipeline corrects for the variable composition of the initial libraries and for the total read count differences between the NGS flow cells.

All libraries were then injected intravenously (i.v.) into adult C57BL/6 mice at a total dose of 1–2 × 10^12^ vg per mouse, corresponding to ~1 × 10^10^ vg per capsid variant. After 1–2 weeks, the mice were sacrificed and the following organs and cells were harvested: abdominal aorta, thoracic aorta, blood cells, biceps, brain, colon, diaphragm, duodenum, eye, brown fat, white fat, heart, inner ear, kidney, liver, lung, ovaries, pancreas, quadriceps femoris, spleen, and stomach. From the mice injected with the third library, we also extracted various immune cells from lymph nodes and the spleen based on their surface expression of CD3 (T lymphocytes), CD19 (B lymphocytes), CD11b (macrophages), or CD11c (dendritic cells). For this reason, whole spleens were not analyzed from mice injected with the third library. Similarly, we did not analyze whole brains because they were also dissected to prepare individual cell types. From these tissues and cells, DNA and RNA was extracted and deep sequenced. For the second and third libraries, we additionally performed qPCR to determine vg per diploid genome (vg/dg) in each tissue (Supplementary Fig. 2a), which permitted to calculate the specificity of a given capsid across all tissues. Finally, we calculated so-called *Bαβ* values (see “Methods” for details) that allow for the depiction and comparison of transcript abundance within the same organ or across all tissues; thus, yielding a complete overview of the biodistribution of all variants (Supplementary Fig. 2b).

**Fig. 2:**
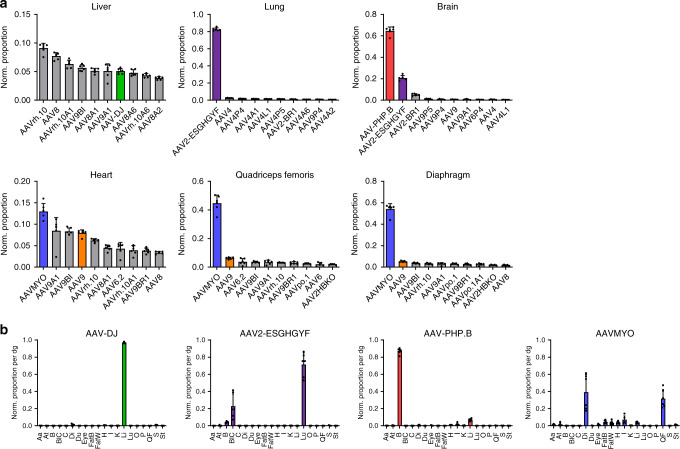
Examples for robust capsids in the liver, lung, brain, and musculature. **a** Top 10 AAV variants in the second library and the shown tissues based on normalized transcriptional efficiency (*V*_*αβ*_). Depicted values are the average from six C57BL/6 J mice with SD. **b** Transcriptional specificity (*T*_*αβ*_) of the shown four capsids as normalized proportion per cell (diploid genome, dg) in abdominal aorta (Aa), thoracic aorta (At), brain (B), blood cells (BlC), colon (C), diaphragm (Di), duodenum (Du), eye, brown fat (FatB), white fat (FatW), heart (H), inner ear (I), kidney (K), liver (Li), lung (Lu), ovaries (O), pancreas (P), quadriceps femoris (QF), spleen (S), and stomach (St). Depicted are average cDNA values from six C57BL/6 J mice with SD. Colors in **a** and **b** highlight the same capsids in both panels. Source data are available in the Source data file.

An overview of all results is provided in Supplementary Figs. 3–13, which show either the top ten capsids per tissue or cell type (efficiency), or the distribution of one capsid across all studied tissues and cells (specificity), both on the DNA or RNA level. In addition, as a specific example, Fig. 1b summarizes the normalized ranking of the best capsids in all three libraries by transcriptional efficiency in the pancreas. The fact that all of the shown lead capsids that were present in all three libraries were in an identical position relative to each other confirms the robustness, and reproducibility of our experimental and bioinformatic workflows. Additional proof of their validity is provided by the data in Fig. 2, which confirms the tissue specificity previously reported for the benchmarks AAV-DJ (liver)^7^, AAV2 modified with peptide ESGHGFY (lung)^4^, and AAV-PHP.B (brain)^3^. Another capsid that we used as benchmark and whose performance was recapitulated in our own data is AAV2-BR1, which consistently ranked among the best candidates in the brain with off-targeting in the lung (Supplementary Figs. 2 and 7), akin to what was described originally^8^. At the same time, we are not surprised that not all previously reported, supposed lead candidates in a given tissue were ranked in our own top ten lists, due to the complexity and diversity of our libraries, and the ensuing chances to find even better capsids that outperform the previous leads. Taken together, the fact that we could validate many of the prior top hits in our screens, whereas we found even better variants in other cases, perfectly illustrates the breadth and capacity of our experimental and bioinformatic workflows.

Intriguing observations were also made regarding the specificity of the 12 wild-type AAVs in our libraries (Supplementary Figs. 7, 8, 11 and 12). While serotypes 1–3, 5–8, rh.10, and 12 predominantly targeted the liver, AAV4 was strongly detargeted from this organ and instead transduced the lung, congruent with former data from others and us^16,17^. Similarly, AAVpo.1 was largely inactive in the liver, but, unlike AAV4 and its derivatives, transduced the musculature, especially the diaphragm and quadriceps femoris. AAV9 exhibited the broadest distribution of all wild-type AAVs and also the highest efficiency in most organs, albeit the majority (~50%) of this capsid still ended up in the liver (Supplementary Fig. 7). Finally, while AAV5 consistently ranked among the most efficient vectors in the liver on the DNA level (Supplementary Figs. 4, 6 and 10), it was outperformed on the mRNA level by numerous other capsids (first library: position 41 of 91; second library: 45 of 82; third library: 124 of 157). The notion that most of the AAV5-encoded genomes fail to express mRNA in the mouse liver is compatible with previous data from others and us and recapitulates (further confirming the validity of our approach) that, at least in mice, AAV5 performs relatively poorly in the liver as compared to other AAV variants^16,17^. This result is further remarkable as it highlights a unique phenotype of AAV5 that may have relevance for emerging clinical data with AAV5 in the human liver^18,19^, while it concurrently exemplifies the species-specific differences (here, mouse versus human) of AAV transduction. Moreover, it showcases the inherent potential of our technology to also describe and dissect fundamental AAV biology.

**Fig. 3:**
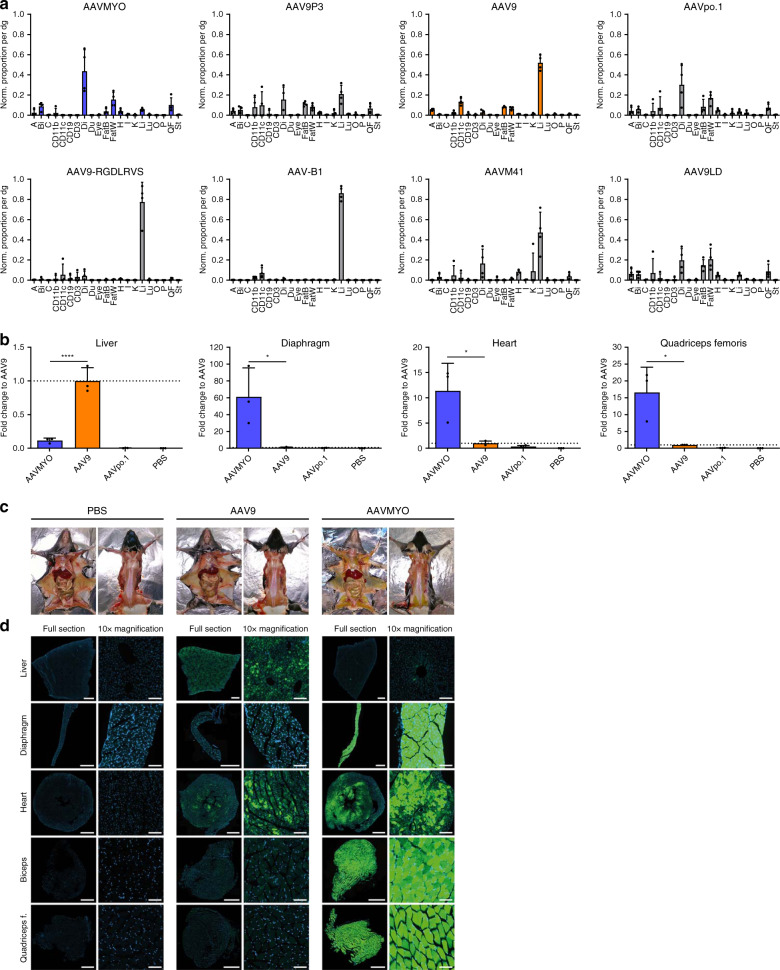
Validation of the myotropic AAV capsid AAVMYO. **a** Transcriptional specificity (*T*_*αβ*_) of the shown capsids from the third library as normalized proportion per cell in aorta (A), biceps (Bi), colon (C), CD11b-, CD11c-, CD19-, or CD3-positive cells, diaphragm (Di), duodenum (Du), eye, brown fat (FatB), white fat (FatW), heart (H), inner ear (I), kidney (K), liver (Li), lung (Lu), ovaries (O), pancreas (P), quadriceps femoris (QF), and stomach (St). Depicted are average cDNA values from six C57BL/6 J mice with SD. **b** Comparison of AAVMYO to AAV9 and AAVpo.1 after individual i.v. injection. Shown are relative *eyfp* mRNA quantities in the liver, diaphragm, quadriceps femoris, and heart. AAV9 values were always set to 1 and the others depicted as fold changes. Relative quantities (2^−ΔΔCt^) of viral *eyfp* transcripts were measured via RT-qPCR and a POLR2A housekeeper. Depicted values are the average of three C57BL/6 J mice with SD. Colors in **a** and **b** highlight the same capsids in both panels; *p < 0.05, **p < 0.01, ***p < 0.001, and ****p < 0.0001 (one-way ANOVA with Tukey’s multiple comparison test). **c** Representative images of dissected C57BL/6 J mice (left: ventral; right: dorsal position) that were i.v. injected with 5 × 10^11^ vg per mouse and sacrificed two weeks later. **d** Representative 10 µm cryosections (*n* = 8 replicates for AAV9, AAVMYO and *n* = 3 for PBS) of the liver, diaphragm, heart, biceps, and quadriceps femoris of the mice from **c**. Direct EGFP fluorescence was detected (green) together with the DAPI signal (blue). Scale bars are 1 mm (full sections) or 100 µm (10× magnifications). Exposure settings were normalized to the liver of the AAV9 group. Source data are available in the Source data file.

**Fig. 4:**
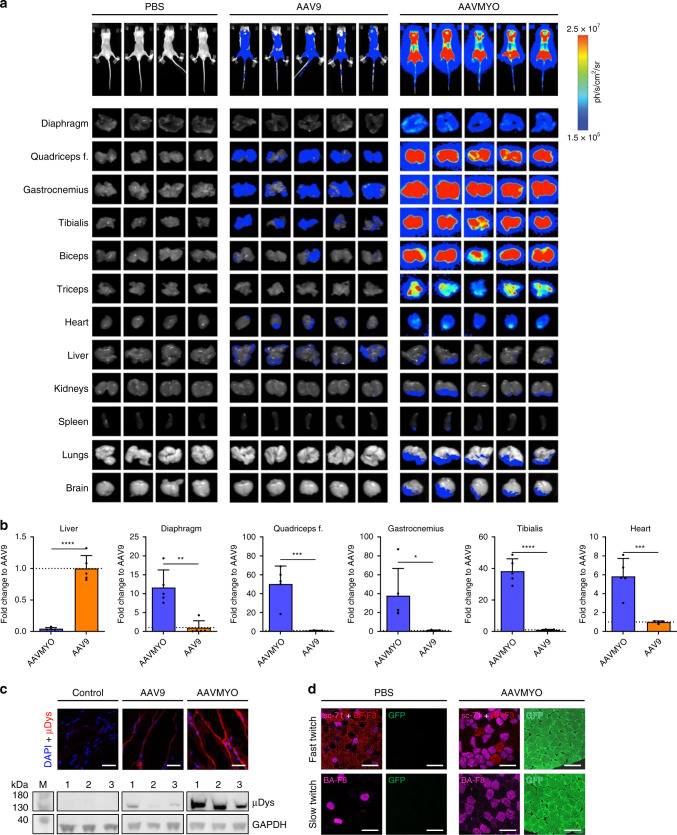
Additional validation of AAVMYO. **a** Shown on top are whole-body images of luciferase expression in CB17-SCID mice 4 weeks after i.v. injection with the shown vectors. Shown underneath are images of luciferase expression in the indicated organs from the same mice, harvested 1 week later. **b** Shown are fold-changes of luciferase mRNA expression of each organ as determined by qRT-PCR in the AAVMYO cohort versus the AAV9 group. Depicted values are the average of the five CB17-SCID mice from panel a with SD; *p < 0.05, **p < 0.01, ***p < 0.001, and ****p < 0.0001 (unpaired two-tailed *t* test). **c** Comparison of µDys expression in quadriceps femoris sections (*n* = 4 replicates for all groups) and protein lysates of *mdx* mice 4 weeks after i.v. injection of 2 × 10^11^ vg (for IHC) or 1 × 10^12^ vg (for western blot) of a control vector (AAVMYO expressing Firefly luciferase), or of AAV9 or AAVMYO encoding µDys. **d** Staining of muscle fibers type I (BA-F8), type IIa (sc-71), type IIb (BF-F3), and GFP in quadriceps femoris sections of C57BL/6 mice (*n* = 4 replicates for all groups) injected with 5 × 10^11^ vg of AAVMYO. Scale bars for **c** and **d** are 25 and 50 µm, respectively. Source data are available in the Source data file.

Likewise, our peptide-modified or shuffled capsids also displayed interesting patterns. For instance, many of the AAV4 peptide-displaying mutants showed a high efficiency in the lung, but not the liver, comparable to wild-type AAV4 (Supplementary Fig. 3). This suggests that the sequences or domains in the AAV4 capsid responsible for the prominent lung tropism after i.v. injection are located outside of variable region VIII that carries the peptide insertions, highlighting the usefulness of our technology to interrogate and dissect basic AAV biology. Another intriguing example are peptides P4 and P5 that, especially when juxtaposed with AAV9, were among the best expressing variants in the brain, albeit they (like all other capsids) were surpassed by AAV-PHP.B^3^. Clearly, though, the most notable discovery in this study is the superior efficiency and specificity of capsid AAV9P1 (from hereon called AAVMYO) in the entire musculature, comprising skeletal muscle, diaphragm, and heart, where it outperformed all other 156 capsids in the second and third libraries (Figs. 2–4). This includes one of the current gold standards in systemic muscle gene therapy, namely AAV9^20^, which was consistently ~10.6-, ~7.2-, or ~1.5-fold inferior to AAVMYO on the RNA level in the diaphragm, quadriceps femoris, or heart, respectively (Fig. 2a and Supplementary Fig. 9). The myospecific properties of AAVMYO are illustrated by the fact that >70% of its associated barcodes were detected in all muscle types in the second library screen (Fig. 2b). This was confirmed with the third library that included four additional benchmarks reported to efficiently transduce muscle types following systemic delivery, i.e., AAVM41^5^, AAV-B1^21^, AAV9LD^22^, and AAV_587_MTP^9^. Moreover, we added two other AAV9 mutants displaying a peptide similar to P1 (RGDLGLS), i.e., AAV9P3 (RGDAVGV) and AAV9-RGDLRVS^23^. As shown in Fig. 3a and Supplementary Fig. 11, none of these benchmarks or variants could match the in vivo efficiency and specificity of AAVMYO. Preliminary data show that AAYMYO also outperforms AAVrh.74 in a direct comparison, which is notable considering that like AAV9, AAVrh.74 is currently under clinical evaluation for muscle gene transfer^24^.

To rule out that these results were influenced by capsid competition within the libraries, we individually injected mice i.v. with 1 × 10^11^ vg of AAVMYO, AAV9, or AAVpo.1, and 1 week later analyzed *eyfp* mRNA expression (Fig. 3b). Remarkably, the improvement of AAVMYO over AAV9 was reproduced and even more pronounced in this separate analysis, with 61-, 17-, or 11-fold increases in the diaphragm, quadriceps femoris, or heart, respectively. Concurrently, AAVMYO was nine-fold detargeted from the liver compared to AAV9, further confirming the data from the bulk analysis.

To validate these results on the protein level, we injected mice with 5 × 10^11^ vg of each vector expressing an eGFP reporter from a CMV promoter and 2 weeks later harvested muscle and liver for histology. Surprisingly, during dissection of the mice, we could already detect eGFP expression by naked eye and in daylight in the AAVMYO-injected animals, but not the AAV9 cohort (Fig. 3c). The superiority of AAVMYO is even more evident in the exemplary images of the histological analysis of eGFP expression in single tissues (Fig. 3d and Supplementary Fig. 14; note that these two figures show the same sections, but the exposure settings were normalized to the liver of the AAV9 group in Fig. 3d and Supplementary Fig. 14d, e, while they were normalized to the diaphragm of the AAVMYO group in Supplementary Fig. 14b–c). Strikingly, i.v. delivered AAVMYO completely transduced the diaphragm, biceps, and quadriceps femoris, as well as most of the heart. In contrast, barely any signal was detected in the liver, confirming the concurrent muscle specificity and liver detargeting (LD) of this unique AAV capsid. Compared to AAVMYO, the AAV9 signals were much weaker overall, especially in the diaphragm and skeletal muscle, whereas this wild-type capsid robustly transduced the liver.

To independently confirm these results with a third reporter and in a second mouse strain, we packaged a Firefly luciferase gene under the muscle-specific SPc5-12 promoter into AAV9 or AAVMYO, and i.v. injected CB17-SCID mice with 4 × 10^10^ vg per mouse. This particular mouse strain was also chosen to remain consistent with previous and ongoing muscle gene transfer experiments in our (T.V. and M.C.) laboratory. Measurement of luciferase expression 4 weeks later in the whole body, and in extracted organs revealed substantially higher expression in all muscle types in the AAVMYO group (Fig. 4a). Quantification of the luciferase mRNA expression showed a 11.6-, 37.8- up to 50.1-, or 5.8-fold increase for AAVMYO over AAV9 in the diaphragm, various skeletal muscle types, or heart, respectively, and a 23.2-fold detargeting in the liver (Fig. 4b and Supplementary Fig. 15).

We note that the extent of the increases with AAVMYO over AAV9 slightly differs between the experiments in Fig. 3 versus Fig. 4, which is, however, most likely due to the use of two different mouse strains and different doses (both were optimized for each experimental setting).

Lastly, we verified the superiority of AAVMYO with yet another transgene, i.e., micro-dystrophin (µDys), by i.v. injecting *mdx* mice with 2 × 10^11^ vg and 1 × 10^12^ vg. Also in this case, we observed a higher expression from AAVMYO after 4 weeks, as compared to AAV9 by immunofluorescence (Fig. 4c top) and western blotting (Fig. 4c bottom). In addition, we were excited to find that AAVMYO transduces multiple types of muscle fibers, i.e., types I (slow-twitch), IIa and IIb (both fast-twitch), highly effectively (Fig. 4d), which, if translatable to humans, broadens its therapeutic index and the range of muscle disorders for which it can be employed.

In recent years, the discovery and development of clinically relevant AAV vectors has been considerably accelerated by the advent of high-throughput techniques for generation and screening of synthetic capsids with advanced properties. A particularly important breakthrough was achieved with the introduction of NGS methodology into the AAV field, and with the first demonstrations of its value by others and us to characterize AAV capsid variants in vivo, by sequencing of peptide insertions^4^, capsid-, insert-, or library-specific barcodes^2,22,25–28^, or entire *cap* genes^29^. Nonetheless, most of these reports share a major caveat, namely, their restriction to tracking solely on the DNA level. Since delivery and expression of AAV-encoded DNA do not automatically correlate, this raises concerns that preferred capsid variants that mediate high transgene expression from low copy numbers will be missed. In the present study, this is best exemplified with AAV5, which appeared as a lead candidate in the liver on the DNA level, while it was in fact very inefficient on the transcriptional level. A recent study also recognized this concern and suggested to use vector-encoded, RNA polymerase III promoter-driven noncoding RNAs for co-tracking on the DNA and RNA level^30^. However, because proof-of-principle was merely provided with a single capsid (AAV2) and in two tissues, the potential for in vivo stratification of larger libraries in an entire organism remained to be explored.

These concerns do not apply to the advanced experimental and bioinformatic pipeline for AAV barcoding and NGS tracking that we introduced and validated here, owing to its combination of pivotal features: (1) barcode insertion in the 3′ untranslated region of the AAV vg, permitting concomitant, qualitative, and quantitative tracking on the DNA and RNA levels; (2) design and use of comprehensive normalization strategies that allow for both, intra- and inter-tissue/-cellular comparisons of capsid performance; (3) use of pre-selected AAV capsids to assemble focused, secondary libraries, rather than randomized and often non-functional variants as in traditional primary AAV evolution strategies^1^; and (4) inclusion of a wide collection of known benchmarks in various tissues, to enable a fair and proper evaluation of in vivo efficiencies and specificities in the same animal(s), and under identical conditions.

The value of these combined assets is best illustrated by our discovery of the extraordinary features of the AAVMYO capsid, yielding transduction of the entire musculature following systemic delivery of moderate doses. By juxtaposing high efficiency and high specificity in the skeletal muscle, heart and diaphragm with pronounced detargeting from the liver (and other organs), AAVMYO is a prime candidate for preclinical development as a vector for gene therapies of human disorders that affect various muscle types and/or different muscle fibers, such as Duchenne muscular dystrophy, Pompe disease, or X-linked myotubular myopathy (XLMTM). In particular, the last example drastically illustrates the urgent need for a next generation of muscle-targeted and concurrently liver-detargeted AAV vectors, such as AAVMYO that work from peripheral administration of low doses, considering recent dire events in the ASPIRO gene therapy trial, in which two children affected by XLMTM passed away after delivery of high doses (3 × 10^14^ vg per kg) of AAV8, potentially related to off-target liver dysfunction (https://clinicaltrials.gov/ct2/show/NCT03199469; https://myotubulartrust.org/audentes-therapeutics-letter-23-june-2020/).

To this end, we note highly encouraging emerging data from our lab implying that AAVMYO may preserve its combination of specificity for the musculature and LD (as compared to AAV9) in non-human primates, too, suggesting that it may be translatable to larger species and ideally humans as well. Our optimism is fueled by our findings that, unlike AAV-PHP.B^3,31^, the properties of AAVMYO are conserved across multiple mouse strains and are predominantly mediated by the P1 peptide, as evidenced by preliminary data that P1 display on 14 different AAV capsid backbones largely preserves its functionality. The latter is further encouraging with respect to future clinical use as it allows to harness synthetic capsid scaffolds that were engineered to, e.g., avoid anti-AAV antibody neutralization. This is particularly important considering that a major and unique strength of AAVMYO is its ability to target the entire musculature from peripheral delivery, making it essential to minimize or avoid adverse immune reactions to circulating AAVMYO particles. While the sum of our present mouse data does not suggest an enhanced transduction of lymphatic tissues or cells with AAVMYO as compared to AAV9, we consider it pivotal to carefully monitor humoral and cellular immune reactions against AAVMYO in future preclinical work in higher species. If observed and if needed, these could then be modulated, for instance, by the use of IgG-cleaving endopeptidases to overcome pre-existing anti-AAV(MYO) antibodies, as most recently suggested by the Mingozzi lab^32^, or, as noted above, by direct capsid engineering.

As demonstrated in Supplementary Fig. 16, AAVMYO can also be produced to high titers akin to its parent AAV9 (that forms the basis of the FDA-approved gene therapeutic Zolgensma) and is compatible with all purification protocols including affinity columns, which shows that AAVMYO is not limited by manufacturing, and thus further increases its translational value.

In the future, it will be very interesting to study and dissect the biology underlying the extreme in vivo biodistribution profile of AAVMYO that we consistently observed in multiple mouse strains in this work, as well as in non-human primates in ongoing work. As noted above, a major contributing factor is certainly the P1 peptide itself, but it is eventually the specific juxtaposition with AAV9 and the insertion at a distinct position in the capsid^15^ that yields the combination of superior muscle on-targeting and liver de-targeting. In this regard, it is crucial to point out that while P1 has been reported before^33^, this was in a completely different context, i.e., AAV2 peptide display and an ex vivo screen in tumor cells derived from a breast cancer mouse model. Equally important to note is that the in vivo phenotype of AAVMYO that we observed here was impossible to predict from our recent ex vivo evaluation of this capsid (and others) in cultured cells^15^, and is thus a surprising finding. Owing to the lack of precedence for such a high and concomitant in vivo specificity of an AAV capsid in skeletal muscle, heart, and diaphragm after systemic administration, and because of the generally limited understanding of AAV biology, the dissection of the exact mechanisms will require extensive additional work. Still, we can already speculate at this point based on multiple lines of evidence and data.

A first intriguing possibility is that AAVMYO’s properties are either caused by an increased on-target specificity in all muscle types, or, additionally or alternatively, by a decreased activity in the liver. The latter acts as primary target and sink for most AAV variants in vivo, hence a de-targeting from this organ would give an AAV capsid/vector more time to circulate in the blood stream and to eventually transduce other tissues, such as the musculature. In the case of AAVMYO, this possibility seems unlikely since it is specifically improved in all muscle types, but nowhere else (with the exception of white fat, see below), which contradicts the expectation if a capsid would circulate for extended periods but transduce nonspecifically. Nonetheless, for more direct evidence, we have recapitulated an informative double-point mutant (P504A/G505A) reported by the Nakai lab in AAV9^22^, and shown by them to detarget this capsid (which is the basis of AAVMYO, too) from the liver. Notably, we could indeed observe an enhanced LD due to this double mutation for the resulting AAVMYO_LD (LD for liver detargeting) capsid as well, as illustrated in Supplementary Fig. 14 (compare Supplementary Fig. 14c to Supplementary Fig. 14b, and Supplementary Fig. 14e to Supplementary Fig. 14d, respectively; these pairs of panels differ in the normalization of the camera settings and are partially reproduced from Fig. 3d, to permit direct comparisons). However, the AAVMYO_LD variant is concurrently also less active in the musculature as compared to the original AAVMYO capsid, which argues against the model that AAVMYO’s biodistribution is merely caused by its pronounced LD (which in itself is highly interesting and worth studying further).

Secondly, the fact that the P1 peptide comprises an RGD motif, which is a known binding partner for integrins^34^, suggests that AAVMYO, as well as other P1-displaying capsids interact with an integrin that is specifically and abundantly expressed on all cells and tissues that are transduced by AAVMYO vectors. A notable candidate and a hypothesis for future work is integrin alpha-7 (ITGA7)/beta-1 that is abundant on all muscle types, and thus matches the predicted profile of a direct AAVMYO ligand on the cell surface. Interestingly, integrins including ITGA7 are also found and upregulated on adipocytes^35^, which could readily explain our recurrent observation in a subset of the mice that AAVMYO also transduces white fat tissue. The model that AAYMYO predominantly gains its muscle specificity from a direct interaction of the P1 peptide sequence with (a) cellular receptor(s) is not only compatible with all our data (including those with other P1-displaying capsids), but also very encouraging as it suggests that P1 can be transferred into other, synthetic capsids that were optimized for additional steps in the transduction pathway and/or are more immunoevasive, all of which will contribute to the next generation of myotropic AAV gene therapy vectors.

Until then, we point out that the pipeline used here for capsid stratification can easily be repurposed to other aims and combined with any other AAV libraries created through different diversification technologies, such as ancestral reconstruction^10^. Important to note again in this context is that, as shown in Fig. 1a, the entire workflow can be accomplished within 6 months starting with the cloning of barcoded AAV vg, or even faster if users recycle our already existing barcoded constructs (which are readily available upon reasonable request), and depending on available computational resources for NGS data analysis. Likewise, in this manuscript, we provide a GitHub link to the bioinformatic resource that permits future users to rapidly establish the analysis pipeline in their labs as well. Accordingly, the entire experimental and bioinformatic workflow described here is neither restricted nor prohibited by time, labor, manpower, or resources, but can instead be implemented easily and quickly in any lab. This is important considering that there are plenty of applications of barcoding technology that have already been exemplified by colleagues and us including in the present work, and that should greatly benefit from the lessons learned here and the resources provided, comprising our scripts for barcode analysis, normalization, and hit ranking. As noted initially and as shown in our work, these manifold applications include barcoding of individual AAV variants or of entire libraries^2,27,28,36^ for primary or secondary screens (the latter is specifically demonstrated here), or to track whole AAV library evolution^26^, as well as the use of barcodes to dissect basic AAV biology^37^. Beyond these applications for AAV capsid evolution, study, and stratification, we are currently exploiting barcoding for in vivo screening of a complex promoter library in an AAV context, and we can readily envision even more uses, such as for comparison of vector doses or delivery routes, or for screening of antibody-evading variants. By itself or when juxtaposed with other complementary in vivo stratification technologies, our pipeline and resources thus promise to rapidly accelerate the design and identification of ideal AAV vectors for human gene therapy on the capsid and genome levels. Nonetheless, we highlight the importance of always thoroughly validating lead candidates on an individual level, as we have done here, to qualitatively and quantitatively control the data obtained by massively parallel approaches. Already, the myotropic AAVMYO capsid described here and the CNS-tropic variants reported in the accompanying work by Dehler and colleagues illustrate the tremendous potential of high-throughput, barcode-based, secondary screening technologies combined with meticulous validation experiments, and they will hopefully encourage other users to harness and adapt these advanced experimental–bioinformatic methodologies for their own agenda as well.

## Methods

### AAV helper plasmids

Helper plasmids co-encoding the AAV2 *rep* gene together with selected *cap* genes were based on a standard AAV helper construct^2^. Depending on the *cap* gene variant, they were already present in our laboratory from former work or created de novo through DNA family shuffling, insertion of peptide-encoding oligonucleotides, gene synthesis, and/or overlap-extension PCR, using standard molecular biology protocols. All final constructs were verified through sequencing of the *cap* gene. Further details on each of the 183 variants used in this work, including full plasmid maps are available from the authors upon request.

### Cloning of barcoded AAV genomes

For the generation of barcoded AAV reporter plasmids, oligonucleotide 5′-TGA CGT CTC TGC TCN NNN NNN NNN NNN NNC AGG CGA GAC GTG ACA CTG C-3′ comprising a stretch of 15 randomized nucleotides (N, the barcode) flanked by two Esp3I restriction sites was ordered from Merck KGaA. Synthesis of the second strand was performed in a 50 µl PCR reaction, including 0.5 µl Phusion Hot Start II DNA polymerase, 10 µl Phusion HF buffer, 1 µl dNTPs (10 mM stock), 1.5 µl dimethyl sulfoxide (all Thermo Fisher Scientific), 0.5 µl reverse primer 5′-GCA GTG TCA CGT CTC GCC TG-3′ (100 µM stock), and, as template, 0.5 µl of the barcode oligonucleotide (100 µM stock). Cycling conditions were 10 s at 98 °C, followed by 30 cycles of 98 °C for 10 s, 70 °C for 30 s, and 72 °C for 5 s, and ending with 5 min at 72 °C. After a subsequent PCR cleanup (DNA Clean & Concentrator-5, Zymo Research), a five-fold molar excess of double-stranded barcode oligonucleotide was mixed with one molar amount of plasmid pscAAV-CMV-EYFP-ccdB-BGHpolyA. The latter is a derivative of a recently reported^15^ self-complementary AAV vector plasmid from our group expressing a YFP reporter under a CMV promoter, and carrying a *ccdB* suicide gene flanked by Esp3I sites in the YFP 3′ untranslated region. Next, 1 µl 10 mM ATP (Merck KGaA), 1 µl 10 mM dithiothreitol, 1 µl 10× Tango buffer, 0.75 µl Esp3I (all Thermo Fisher Scientific), 1 µl T4 DNA ligase (New England Biolabs), and nuclease-free H_2_O (up to a total volume of 10 µl) were added. A Golden Gate cloning reaction was carried out by incubating the mixture for 5 min at 37 °C, followed by 5 min at 16 °C. These two steps were repeated 19 times before heat inactivating the mixture for 20 min at 65 °C. During this reaction, the *ccdB* gene is replaced with a barcode (one per plasmid). After transformation into electrocompetent MegaX DH10B™ T1R Electrocomp™ cells (Invitrogen), the integrity of the AAV ITRs was confirmed in individual clones by restriction digest with PstI and XmaI, and the barcode of positive clones was sequenced. Barcodes whose length differed from 15 nt or that comprised homopolymers longer than 3 nt were excluded. The Hamming distance^38^ of the remaining pool was assessed and sequences that were distinguished from all other barcodes in at least five positions were kept, yielding the 159 barcodes listed in Supplementary Table 2. All oligonucleotides are listed in Supplementary Table 3.

### AAV production

Production, purification, and titration of the barcoded AAV vectors and of the individual AAV vectors for validation experiments was performed using standard technology, including AAV-293 cells (Stratagene/Agilent), as well as the use of standard qPCR for titration and 1× PBS as final buffer^39,40^. To generate the first barcoded AAV library, two 15 cm dishes were used for separate production of each variant. Cell lysates were subsequently pooled, and this pool was then purified on a single cesium chloride density gradient. For the second and third library, each barcoded vector was individually produced and purified on an iodixanol density gradient. Afterward, 1.2 × 10^11^ vg of each vector were pooled and concentrated with an Amicon Ultra-15 (Merck KgaA; used for the first and second library) or Pierce Protein Concentrator (Thermo Fisher Scientific; used for the third library).

### Animals

Seven-week-old female inbred C57BL/6 J mice (Janvier Labs) were used for all in vivo library screens, as well as for the AAVMYO validation experiments except for the luciferase and µDys studies. Mice were kept and handled in accordance with the animal protocols 35-9185.81/G-126/14 and 35-9185.81/G-89/16 that were approved by the Regierungspräsidium Karlsruhe (Germany). All in vivo and ex vivo luciferase imaging procedures were conducted in CB17-SCID mice, and were approved by the institutional animal ethics committee of the Free University of Brussels (VUB; Brussels, Belgium). Husbandry was carried out in individually ventilated Thoren cages that contained Hygienic Animal Bedding (Lignocel). Temperature was maintained at ~21 °C with 50–60% humidity. Animals were fed SsniFF laboratory animal food (ABEDD Vertriebs GmbH, Vienna, Austria) ad libitum. Analysis of µDys expression was performed in 6-week-old male *mdx* mice that were bred in-house and housed in a temperature and humidity controlled room in a specified pathogen-free environment under 12:12 h light/dark cycles. These mice can be obtained at https://www.jax.org/strain/001801 and are published^41^. All procedures involving the use and care of animals were performed according to the Directive 2010/63/EU of the European Parliament and the German animal protection code. Permission was granted by local authorities (V 242-12956/2018).

### In vivo AAV capsid screening

Mice were i.v. injected via the tail vein with the barcoded AAV library at ~1 × 10^12^ vg per mouse in a total volume of 150–200 µl 1× PBS. Note that the amount of injected AAV particles was identical in all in vivo screening experiments despite the minor variation in total injection volume. One to 2 weeks later (first library: 15 days, second and third libraries: 8 days), abdominal aorta, thoracic aorta, brain, biceps, blood cells, colon, diaphragm, duodenum, eye, brown fat, white fat, heart, inner ear, kidney, liver, lung, ovaries, pancreas, quadriceps femoris, spleen, and stomach were harvested, and tissue pieces were submerged in RNAlater solution (Thermo Fisher Scientific) before storing at −20 °C.

### MACS-based isolation of immune cells

Isolation of CD3ε-, CD11b-, CD11c-, and CD19-positive cells was performed by harvesting the mandibular, accessory mandibular, subiliac, proper axillary, accessory axillary, and medial iliac lymph nodes, as well as the spleen. Tissues were transferred to a 70 µm strainer and homogenized with a plunger. After washing the strainer with MACS buffer (1× PBS, 0.5% bovine serum albumin (BSA), and 2 mM EDTA), the resulting cell suspension was centrifuged at 1000 r.c.f. for 5 min. The supernatant was aspirated and the pellet resuspended in 10 ml RBC lysis solution (Miltenyi Biotec) before incubating for 5 min at room temperature. Cells were centrifuged again at 1000 r.c.f. for 5 min and resuspended in 1 ml MACS buffer, yielding ~1 × 10^8^ cells per ml. Next, the cell suspension was split into two 500 µl fractions, to which 100 µl CD11c or CD11b MicroBeads (Miltenyi Biotec) were added, respectively. The following steps were carried out according to the manufacturer’s instructions for these beads. The flow-through fraction of both purifications was kept and used to isolate CD19- and CD3-positive cells, respectively, by following the manufacturer’s instructions. Purified cells were counted and subsequently pelleted before freezing in liquid nitrogen for storage at −80 °C.

### Tissue and cell homogenization

Isolated tissues were removed from RNAlater solution and weighed. Following tissue transfer to Precellys® tubes (Bertin Instruments, one per tissue), 350 µl of RLT buffer with 1% β-mercaptoethanol (QIAGEN) was added for every 10 mg of tissue. Tubes were placed into a Precellys® 24-dual homogenizer and homogenized at 5500 r.p.m. for 20 s. For samples that were insufficiently homogenized, the procedure was repeated. Cell pellets from the MACS purification were resuspended in 300 µl RLT buffer with 1% β-mercaptoethanol for every 1 × 10^6^ cells and incubated for 5 min at room temperature. Lysates were transferred to a QiaShredder tube (QIAGEN) and centrifuged at 13,000 r.c.f. for 2 min.

### DNA/RNA extraction and cDNA synthesis

The 5PRIME Phase Lock Gel tubes (Quantabio) were centrifuged at 16,000 r.c.f. for 30 s to collect the gel at the bottom of the tube. Afterward, 400 µl phenol/chloroform/isoamyl alcohol (25:24:1, Merck KGaA) was added. Tissue lysates were thawed and subsequently centrifuged at 3200 r.c.f. for 4 min to pellet remaining debris. A total of 400 µl of tissue lysate was transferred to a prepared 5PRIME Phase Lock Gel tube and shaken vigorously for 15 s. After centrifugation at 16,000 r.c.f. for 5 min, 400 µl chloroform/isoamylalcohol (24:1, Merck KGaA) was added, and the tubes were again shaken vigorously for 15 s. Tubes were incubated for 3 min at room temperature before centrifuging at 16,000 r.c.f. for 5 min. Next, 350 µl of the aqueous phase and the 300 µl RLT lysate of the immune cells were used to isolate DNA and RNA with the Allprep DNA/RNA 96 Kit (QIAGEN). Contaminating genomic (g)DNA was removed from the RNA fraction by using the RNase-free DNase I Set (QIAGEN). The digest was performed on the RNA-binding silica column and additionally with the RNA eluate in solution, as we noticed that a single on-column DNase I digestion is insufficient to remove all residual gDNA (Supplementary Fig. 17). After complete removal of contaminating DNA, DNase I was heat inactivated for 10 min at 75 °C. gDNA-free RNA was reverse-transcribed into cDNA by using the High-Capacity cDNA Reverse Transcription Kit (Thermo Fisher Scientific).

### NGS of amplified barcodes

The barcode region of the cDNA and gDNA samples was amplified by PCR in a 50 µl reaction comprising 0.5 µl Phusion Hot Start II DNA Polymerase (Thermo Fisher Scientific), 10 µl 5× Phusion HF buffer, 1 µl dNTPs (10 mM stock), 0.25 µl forward primer 5′-ATC ACT CTC GGC ATG GAC GAG C-3′ (100 µM stock), 0.25 µl reverse primer 5′-GGC TGG CAA CTA GAA GGC ACA-3′ (100 µM stock), and 25 ng of cDNA or gDNA as template. Cycling conditions were 30 s at 98 °C, followed by 40 cycles of 98 °C for 10 s and 72 °C for 20 s, and a final 5 min step at 72 °C. The PCR reaction was subsequently cleaned up with the MagMAX Express-96 Magnetic Particle Processor and Agencourt AMPure XP beads (Beckman Coulter, 100 µl per 50 µl PCR reaction). The outcomes of the PCRs and the DNA concentrations were analyzed on a Fragment Analyzer using the Standard Sensitivity NGS Fragment Analysis Kit (Advanced Analytical). To prepare the amplicon sequencing library, the Ovation Library System for Low Complexity Samples Kit (NuGEN Technologies, Inc.) was used to process 20–30 ng of amplicon DNA per sample. Results were monitored by running the processed samples on a Fragment Analyzer with the Standard Sensitivity NGS Fragment Analysis Kit. Quantification of the DNA concentration of the samples was performed with the Quant-iT PicoGreen dsDNA Assay Kit (Thermo Fisher Scientific). Based on the DNA concentrations, a 2 nM dilution was prepared for each sample with Illumina resuspension buffer containing 0.1% Tween20. A total of 10 µl of every 2 nM dilution with a unique reverse adaptor, which permits multiplexing on the flow cell, were mixed to generate the sequencing library pool. To denature the library fragments, 5.3–6.0 µl of the library pool were used and filled up to 10 µl with Illumina resuspension buffer containing 0.1% Tween20. Next, 10 µl of 0.2 M sodium hydroxide were added, vortexed, and incubated for 5 min at room temperature to denature the DNA strands. For neutralization, 10 µl of 200 mM Tris-HCl, pH 7.0 were added and samples were vortexed. The denatured library pool dilution was filled to 1 ml with 970 µl of prechilled HT1 buffer (Illumina, Inc.) and mixed, before 117 µl were combined with 1183 µl of prechilled HT1 buffer. Then, 2 µl of 20 pM PhiX control were spiked in. The final library pool dilution (1 pM) was vortexed thoroughly, spun down, and loaded into a NextSeq500 cartridge (Illumina, Inc.). Screen instructions were followed to start the NextSeq500. Read 1 was set to 84 and index 1 to 8.

### Detection of vector genomes by qPCR

Using the extracted DNA from the tissues and cells, a 30 µl qPCR reaction was performed including 15 µl QuantiFast PCR Master Mix, forward primer 5′-GAG CGC ACC ATC TTC TTC AAG-3′, reverse primer 5′-TGT CGC CCT CGA ACT TCA C-3′, and probe 5′-ACG ACG GCA ACT ACA-3′ (60× mix in total, of which 0.5 µl were used) and 14.5 µl sample (75 ng) to detect *eyfp*-containing vector genomes. GAPDH primer/probe mix Mm00186825_cn (Thermo Fisher Scientific) was utilized to determine the copy number of the housekeeper gene. In both cases (*eyfp* or *GAPDH*), 10 µl were transferred to one well of a 384-well plate and subjected to the following qPCR cycling conditions: 2 min at 50 °C and 10 min at 95 °C, followed by 40 cycles of 95 °C for 15 s and 60 °C for 1 min. Resulting *GAPDH* values were divided by two to obtain the number of dg, and *eyfp* copy numbers were divided by the amount of dg, resulting in vector genomes per diploid genome (vg/dg). These so-called *G*_*β*_ values were used for normalization of the sequencing data.

Biodistribution of the AAV constructs containing the *Luc2* luciferase gene was studied by quantifying *Luc2* transgene copy numbers in the different organs and tissues. After DNA extraction, 100 ng gDNA from each sample was subjected to qPCR using *Luc2*-specific primers (forward primer 5′-CCC ACC GTC GTA TTC GTG AG-3′ and reverse primer 5′-TCA GGG CGA TGG TTT TGT CCC-3′), yielding a 206 bp amplicon. The qPCR results were expressed as mean AAV genome copy number per gDNA (vg/dg). Known copy numbers (10^2^–10^7^) of the corresponding plasmid were serially diluted and used to generate a standard curve.

### NGS data normalization

For NGS data processing, we expanded on a previous approach by the Zolotukhin lab^25^, and implemented a Python 2.7 script that uses the demultiplexed reads from the sequencer and searches for the known 15 nt-long barcode sequences. The output file lists the unknown sequences, as well as the variant-assigned barcodes with their corresponding read counts. A second Python 2.7 script utilizes the output files from the first script and performs a multistep normalization procedure, which corrects for (i) the variations in the total read counts of each flow cell, (ii) the unbalanced composition of the initial viral injection mixture, and (iii) different efficiencies of the AAV library in the analyzed tissues. In the first step, the script normalizes the read counts *R* of all variants *α* in tissue *β* to the sum of all variants *α* in *β*, to obtain the proportion *P*_*αβ*_. The second step corrects for the uneven composition of the library, by dividing *P*_*αβ*_ by the NGS-determined proportion *L*_*α*_ of each variant *α* in the initial library that was used for the injection, resulting in P*_αβ_. In the third step, P*_αβ_ is multiplied by the qPCR-determined vg/dg (also called *G*_*β*_), to enable the comparison of one variant *α* over all analyzed tissues *β*. The obtained value is called *B*_*αβ*_. In this work, *B*_*αβ*_ values are depicted as heat maps visualizing the differences of all variants *α* in all tissues *β*. Moreover, *B*_*αβ*_ values can also be shown as proportion of the sum over *α* (resulting in *V*_*αβ*_) or *β* (resulting in *T*_*αβ*_) of *B*_*αβ*_. Here, *V*_*αβ*_ values are illustrated as bar plots that demonstrate the proportion of all variants *α* in one tissue *β*, and therefore exemplify the efficiency of the individual vectors. Bar plots using *T*_*αβ*_ values show the proportion of one variant *α* in all tissues *β*, allowing for an analysis of vector tissue specificity. The complete mathematical workflow is also illustrated in Supplementary Fig. 18.

### Reverse-transcription (RT-)qPCR

Vectors encoding a CMV promoter-driven *egfp* reporter gene in capsids AAVMYO, AAV9, or AAVpo.1 were injected into three C57BL/6 J mice through the tail vein, each at a dose of 1 × 10^11^ vg per mouse. Control mice were injected with 1× PBS. All mice were kept for 1 week before harvesting diaphragm, quadriceps femoris, heart, and liver. RNA that had been extracted with aforementioned isolation protocols was analyzed by RT-qPCR with 15 µl QuantiFast PCR Master Mix, forward primer 5′-GAG CGC ACC ATC TTC TTC AAG-3′, reverse primer 5′-TGT CGC CCT CGA ACT TCA C-3′, and probe 5′-ACG ACG GCA ACT ACA-3′ (60× mix in total, of which 0.5 µl were used), 13.5 µl sample (50 ng) and 1 µl H_2_O to detect *eyfp*-containing viral transcripts. POLR2A primer/probe mix Mm00839502_m1 (Thermo Fisher Scientific) was used to quantify the housekeeper transcripts. The 2^−ΔΔCt^ values were calculated in relation to POLR2A and the AAV9 cohort.

For the measurement of luciferase mRNA expression, total RNA was extracted from different tissues of mice injected with the various AAV vectors using a Qiagen AllPrep DNA/RNA purification kit (QIAGEN). Subsequently, 100 ng of total RNA from each sample was subjected to reverse transcription (RT) using the SuperScript IV cDNA synthesis kit (ThermoFisher Scientific). Next, a cDNA amount corresponding to 100 ng of total RNA was amplified by qPCR on an ABI 7700 (Applied Biosystems). To quantify *Luc2* mRNA levels in different tissues, RT-qPCR analysis was performed using *Luc2*-specific primers, as mentioned above. *Luc2* levels were normalized to mRNA levels of the endogenous murine glyceraldehyde-3-phosphate dehydrogenase (*mGapdh*) gene, using primers 5′-TGT GTC CGT CGT GGA TCT GA-3′ and 5′-GCC TGC TTC ACC ACC TTC TTG A-3′, yielding a 82 bp amplicon. Relative mRNA expression levels were calculated using the 2^−ΔΔCt^ formula.

### Histological analysis of native GFP expression in muscle tissues

To validate the tissue specificity of AAVMYO by histology, 6-week-old female C57BL/6 J mice were i.v. injected with 5 × 10^11^ vg per mouse and kept for 2 weeks before harvesting biceps, diaphragm, heart, liver, and quadriceps femoris. Injected vectors expressed *egfp* under the control of a CMV promoter. Tissues were fixed in 4% paraformaldehyde for 15–22 h and subsequently transferred to 30% sucrose solution, in which they were kept until the tissue sank to the bottom of the tube (~6 h). Afterwards, organs were embedded in TissueTek® O.C.T Compound (Sakura Finetek Europe B.V. KvK), frozen on dry ice and stored at −80 °C. A total of 12 µm sections were cut and embedded in ProLong™ Gold antifade reagent containing 4′,6-diamidin-2-phenylindol (DAPI; Thermo Fisher Scientific). Sections were scanned with an Axio Scan.Z1 detecting the DAPI and GFP signals.

### Luciferase imaging

The pscAAV-SPc5-12-Luc2-polyA AAV vector construct expressing Firefly luciferase under the muscle-specific SPc5-12 promoter, as well as conditions for in vivo imaging of transduced mice have been reported recently (Tulalamba et al., in press). Briefly, 4-week-old CB17/IcrTac/Prkdc^scid^ mice were injected i.v. with purified AAV9 or AAVMYO expressing a *Luc2* reporter gene under the SPc5-12 promoter at a dose of 4 × 10^10^ vg per mouse. Four weeks later, the mice were injected i.v. with D-luciferin in saline (30 mg/ml) at a dose of 150 mg/kg of body weight, and then subjected to bioluminescence imaging analysis using an in vivo optical imaging system (PhotonImager, Biospace Lab).

For quantitative image analysis of individual organs, mice were euthanized by cervical dislocation within 1 min after D-luciferin administration. Organs were extracted and measured using the PhotonImager optical imaging system. Raw images containing raw data were then analyzed in M3Vision software (Biospace Lab), using the freehand tool to obtain total luciferase signals from each organ. Data were exported in photons (ph)/s/cm^2^/steradian unit and displayed as a pseudo-color overlay onto a gray scale animal image, using a rainbow color scale.

### Micro-dystrophin expression and measurement

Single-stranded AAV vectors encoding µDys were based on a µDys cDNA^42^ that was expressed under the control of the muscle creatine kinase promoter. As control, Firefly luciferase was expressed from the same promoter. At the age of 6 weeks, male *mdx* mice were administered with 2 × 10^11^ AAV9 or AAVMYO vg via tail vein injection (*n* = 3–4 per group). Four weeks post injection, the animals were sacrificed by cervical dislocation. Tissue was harvested, immediately embedded in TissueTek O.C.T. Compound (Sakura Finetek Europe), and frozen for sectioning. Serial 5 µm transverse cryosections were cut from skeletal muscle (quadriceps femoris) and stained with rabbit polyclonal antibody RB-9024-P (1:500 dilution in PBS with 2.5% BSA and 0.05% Triton X-100, Thermo Fisher Scientific) against the dystrophin C-terminus over night at 4 °C, followed by incubation with Alexa Fluor 546-coupled donkey anti-rabbit secondary antibody (A10040, 1:400 dilution, Invitrogen, Thermo Fisher Scientific) along with DAPI (1:1000 dilution, Vector Laboratories) for 1 h at room temperature in the dark. Washing was performed with PBS containing 0.05% Tween20. Following their embedding in FluorSave Reagent (Merck Millipore), the sections were analyzed by fluorescence microscopy using a LSM 800 microscope (Zeiss). For western blotting, skeletal muscle tissue (quadriceps femoris) was transferred into lysis buffer containing 20 mmol/l Tris (pH 7.5), 500 mmol/l NaCl, 12.5% (v/v) glycerol, 10 mmol/l dithriothreitol, 1% (v/v) Nonidet P-40, protease inhibitor cocktail tablets (Roche), as well as phosphatase inhibitor cocktail 2 and 3 (Sigma-Aldrich), and homogenized using an Ultra-Turrax T25 tissue separator (Janke&Kunkel). Cell debris was removed by centrifugation and the concentration of total protein extracted from muscle tissues determined by DC Protein Assay (Bio-Rad Laboratories) according to the manufacturer’s guidelines. Proteins were resolved on 4–12% gradient gels (Life Technologies) and then transferred onto nitrocellulose membranes. Following blocking for 2 h in 3% dry milk prepared in 0.1% TBST at room temperature, membranes were incubated with primary antibodies over night at 4 °C (rabbit polyclonal antibody RB-9024-P against the Dystrophin C-terminus, 1:800 dilution, Thermo Fisher Scientific; or mouse monoclonal antibody against GAPDH, 1:5000 dilution, Sigma-Aldrich) in 3% dry milk in 0.1% TBST. After four washes (10 min each) with 0.1% TBST, membranes were incubated with a suitable horse radish peroxidase-coupled secondary antibody (1:10000, Santa Cruz). Following another four washes, bound antibodies were detected using the ECL-select chemiluminescence kit (GE Healthcare) and visualized on a FluorChem Q imaging system (Biozym).

### Muscle fiber staining

Eight-week-old male C57BL/6 mice were i.v. injected with 5 × 10^11^ vg per mouse of AAVMYO carrying a self-complementary genome encoding a CMV promoter-driven *gfp* reporter. After 2 weeks, quadriceps femoris was harvested and 7 µm thick muscle sections were prepared for immunohistochemistry^43^. In brief, muscle sections were fixed with acetone for 5 min at −20 °C, followed by 5 min washing with PBS. To block nonspecific binding, sections were incubated for 1 h with 10% goat serum (Sigma-Aldrich). Next, BA-F8, sc-71, and BF-F3 (Developmental Studies Hybridoma Bank) were used as primary antibodies to detect the muscle fiber types I, IIa, and IIb, respectively. AAVMYO-induced GFP expression was measured with an anti-GFP-Alexa488 antibody (Cell Signalling). BA-F8, sc-71, BF-F3, and anti-GFP were diluted 1:50, 1:100, 1:100, or 1:500, respectively, in a buffer containing 2.5% BSA and 0.05% Triton X-100, and incubated with the sections overnight at 4 °C in a humidified atmosphere. After a series of washes in PBS containing 0.01% Triton X-100, tissues were treated with corresponding secondary antibodies Alexa Fluor 546 goat anti-mouse IgM, Alexa Fluor 647 goat anti-mouse IgG2b, and Alexa Fluor 647 goat anti-mouse IgG1 (Thermo Fisher Scientific) at a dilution of 1:400 for 1 h at room temperature. After three 10 min washing steps, sections were mounted (Fluoroshied mounting medium, Sigma-Aldrich) and air-dried before imaging using confocal microscopy (LSM 800, Zeiss). A non-injected mouse served as control for anti-GFP immunostaining. To rule out nonspecific secondary antibody binding, we included further controls in which primary antibody incubation was omitted.

### Statistics

Statistical analyses were performed with GraphPad Prism 8 using a one-way ANOVA, with Tukey’s multiple comparison test for Figs. 3b and 4d, and Supplementary Fig. 15b. An unpaired two-tailed *t* test was used for Fig. 4b and Supplementary Fig. 15a. *P* values are defined as <0.05 = *, <0.01 = **, <0.001 = ***, and <0.0001 = ****.

### Reporting summary

Further information on research design is available in the Nature Research Reporting Summary linked to this article.

## Supplementary information

Supplementary Figures, Tables and Discussion

Reporting Summary

## Data Availability

All data generated or analyzed during this study are included in this published article and its Supplementary information files. Raw sequencing data are available via accession code PRJNA557319. The complete nucleotide sequence of AAVMYO can be found under GenBank accession code MN365014. Alternatively, its sequence as well as the complete sequence of the AAVMYO helper plasmid can be directly obtained from the authors upon request. Further details on each of the 183 variants used in this work, including full plasmid maps are available from the authors upon request. Any other relevant data are available from the authors upon reasonable request. Source data are provided with this paper.

## Source data

Source Data

## Code availability

The scripts used for barcode detection and normalization are available via GitHub (https://github.com/JonasWeinmann/AAV-barcode-detection-and-normalization).

## References

[1] Grimm D, Zolotukhin SE (2015). Pluribus unum: 50 years of research, millions of viruses, and one goal-tailored acceleration of AAV evolution. Mol. Ther..

[2] Herrmann AK (2019). A robust and all-inclusive pipeline for shuffling of adeno-associated viruses. ACS Synth. Biol..

[3] Deverman BE (2016). Cre-dependent selection yields AAV variants for widespread gene transfer to the adult brain. Nat. Biotechnol..

[4] Korbelin J (2016). Pulmonary targeting of adeno-associated viral vectors by next-generation sequencing-guided screening of random capsid displayed peptide libraries. Mol. Ther..

[5] Yang L (2009). A myocardium tropic adeno-associated virus (AAV) evolved by DNA shuffling and in vivo selection. Proc. Natl Acad. Sci. USA.

[6] Lisowski L (2014). Selection and evaluation of clinically relevant AAV variants in a xenograft liver model. Nature.

[7] Grimm D (2008). In vitro and in vivo gene therapy vector evolution via multispecies interbreeding and retargeting of adeno-associated viruses. J. Virol..

[8] Korbelin J (2016). A brain microvasculature endothelial cell-specific viral vector with the potential to treat neurovascular and neurological diseases. EMBO Mol. Med..

[9] Yu CY (2009). A muscle-targeting peptide displayed on AAV2 improves muscle tropism on systemic delivery. Gene Ther..

[10] Zinn E (2015). In Silico reconstruction of the viral evolutionary lineage yields a potent gene therapy vector. Cell Rep..

[11] Dalkara D (2013). In vivo-directed evolution of a new adeno-associated virus for therapeutic outer retinal gene delivery from the vitreous. Sci. Transl. Med..

[12] Opie SR, Warrington KH, Agbandje-McKenna M, Zolotukhin S, Muzyczka N (2003). Identification of amino acid residues in the capsid proteins of adeno-associated virus type 2 that contribute to heparan sulfate proteoglycan binding. J. Virol..

[13] Li M (2010). High-efficiency transduction of fibroblasts and mesenchymal stem cells by tyrosine-mutant AAV2 vectors for their potential use in cellular therapy. Hum. Gene Ther..

[14] Limberis MP, Vandenberghe LH, Zhang L, Pickles RJ, Wilson JM (2009). Transduction efficiencies of novel AAV vectors in mouse airway epithelium in vivo and human ciliated airway epithelium in vitro. Mol. Ther..

[15] Börner K (2020). Pre-arrayed pan-AAV peptide display libraries for rapid single-round screening. Mol. Ther..

[16] Grimm D (2003). Preclinical in vivo evaluation of pseudotyped adeno-associated virus vectors for liver gene therapy. Blood.

[17] Zincarelli C, Soltys S, Rengo G, Rabinowitz JE (2008). Analysis of AAV serotypes 1-9 mediated gene expression and tropism in mice after systemic injection. Mol. Ther..

[18] D’Avola D (2016). Phase I open label liver-directed gene therapy clinical trial for acute intermittent porphyria. J. Hepatol..

[19] Miesbach W (2018). Gene therapy with adeno-associated virus vector 5-human factor IX in adults with hemophilia B. Blood.

[20] Yue Y (2008). A single intravenous injection of adeno-associated virus serotype-9 leads to whole body skeletal muscle transduction in dogs. Mol. Ther..

[21] Choudhury SR (2016). In vivo selection yields AAV-B1 capsid for central nervous system and muscle gene therapy. Mol. Ther..

[22] Adachi K, Enoki T, Kawano Y, Veraz M, Nakai H (2014). Drawing a high-resolution functional map of adeno-associated virus capsid by massively parallel sequencing. Nat. Commun..

[23] Varadi K (2012). Novel random peptide libraries displayed on AAV serotype 9 for selection of endothelial cell-directed gene transfer vectors. Gene Ther..

[24] Mendell JR (2019). Gene delivery for limb-girdle muscular dystrophy type 2D by isolated limb infusion. Hum. Gene Ther..

[25] Marsic D, Mendez-Gomez HR, Zolotukhin S (2015). High-accuracy biodistribution analysis of adeno-associated virus variants by double barcode sequencing. Mol. Ther. Methods Clin. Dev..

[26] de Alencastro G (2020). Tracking adeno-associated virus capsid evolution by high-throughput sequencing. Hum. Gene Ther..

[27] Westhaus A (2020). High throughput in vitro, ex vivo and in vivo screen of AAV vectors based on physical and functional transduction. Hum. Gene Ther..

[28] Davidsson M (2019). A systematic capsid evolution approach performed in vivo for the design of AAV vectors with tailored properties and tropism. Proc. Natl Acad. Sci. USA.

[29] Paulk NK (2018). Bioengineered AAV capsids with combined high human liver transduction in vivo and unique humoral seroreactivity. Mol. Ther..

[30] Xu M (2019). High-throughput quantification of in vivo adeno-associated virus transduction with barcoded non-coding RNAs. Hum. Gene Ther..

[31] Hordeaux J (2019). The GPI-linked protein LY6A drives AAV-PHP.B transport across the blood-brain barrier. Mol. Ther..

[32] Leborgne C (2020). IgG-cleaving endopeptidase enables in vivo gene therapy in the presence of anti-AAV neutralizing antibodies. Nat. Med..

[33] Michelfelder S (2009). Successful expansion but not complete restriction of tropism of adeno-associated virus by in vivo biopanning of random virus display peptide libraries. PLoS ONE.

[34] Ruoslahti E (1996). RGD and other recognition sequences for integrins. Annu. Rev. Cell Dev. Biol..

[35] Morandi EM (2016). ITGAV and ITGA5 diversely regulate proliferation and adipogenic differentiation of human adipose derived stem cells. Sci. Rep..

[36] Davidsson M (2016). A novel process of viral vector barcoding and library preparation enables high-diversity library generation and recombination-free paired-end sequencing. Sci. Rep..

[37] Earley LF (2017). Adeno-associated virus (AAV) assembly-activating protein is not an essential requirement for capsid assembly of AAV serotypes 4, 5, and 11. J. Virol..

[38] Hamming RW (1950). Eror detecting and error correcting codes. Bell Syst. Tech. J..

[39] Borner K (2013). Robust RNAi enhancement via human Argonaute-2 overexpression from plasmids, viral vectors and cell lines. Nucleic Acids Res..

[40] Sarcar S (2019). Next-generation muscle-directed gene therapy by in silico vector design. Nat. Commun..

[41] Wagner S (2012). The heart in Duchenne muscular dystrophy: early detection of contractile performance alteration. J. Cell. Mol. Med..

[42] Schinkel S (2012). Long-term preservation of cardiac structure and function after adeno-associated virus serotype 9-mediated microdystrophin gene transfer in mdx mice. Hum. Gene Ther..

[43] Ribaric S, Cebasek V (2013). Simultaneous visualization of myosin heavy chain isoforms in single muscle sections. Cells Tissues Organs.

