# Supplementary material for: Identification of a myotropic AAV by massively parallel in vivo evaluation of barcoded capsid variants
Source: Nat Commun. 2020 Oct 28;11:5432. doi: 10.1038/s41467-020-19230-w (PMC7595228; doi:10.1038/s41467-020-19230-w)
Supplement: Supplementary file 1 — Supplementary Figures, Tables and Discussion [file 41467_2020_19230_MOESM1_ESM.pdf]

**Identification of a myotropic AAV by massively parallel *in vivo* evaluation of barcoded capsid variants**

Weinmann *et al*.

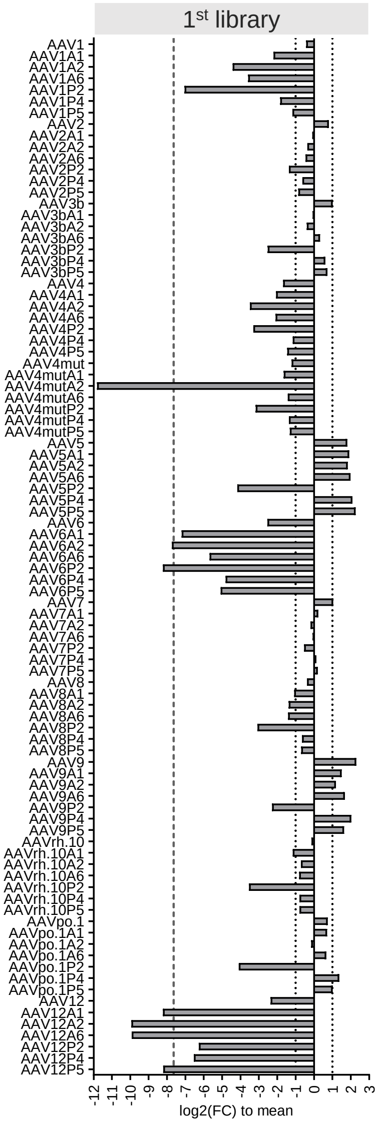


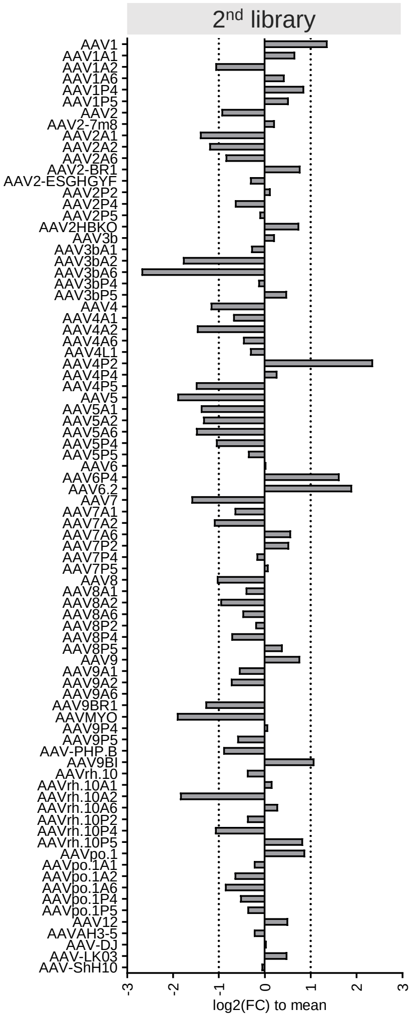


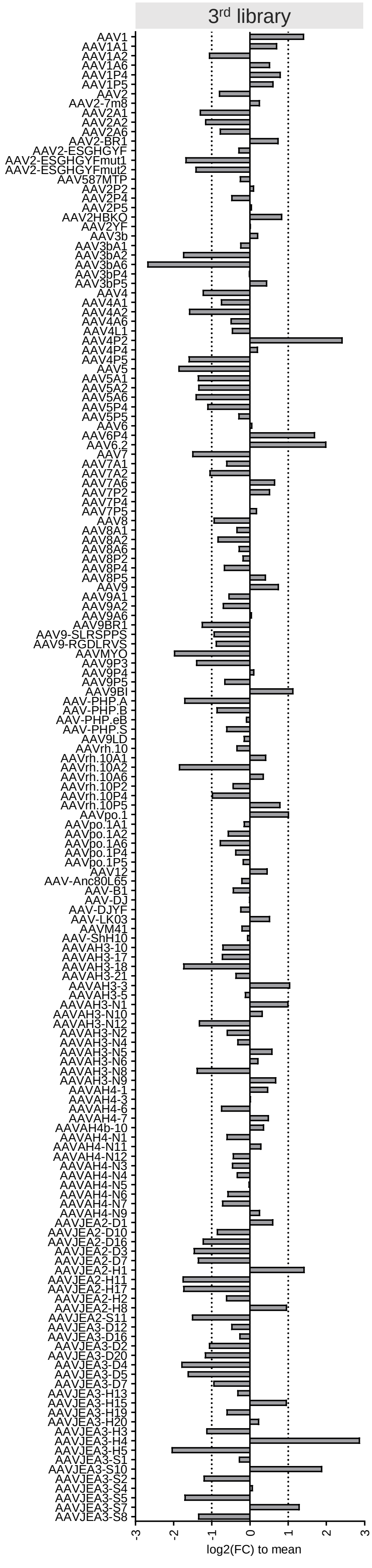


**Supplementary Fig. 1. Composition of 1st, 2nd and 3rd barcoded library.** Shown are log2(FC) values relative to the mean for each AAV variant in the respective library. Negative values indicate under-representation of the respective variant, positive values imply over-representation. Dotted line marks a 2-fold change relative to the mean, dashed line a 200-fold change. FC, fold change.

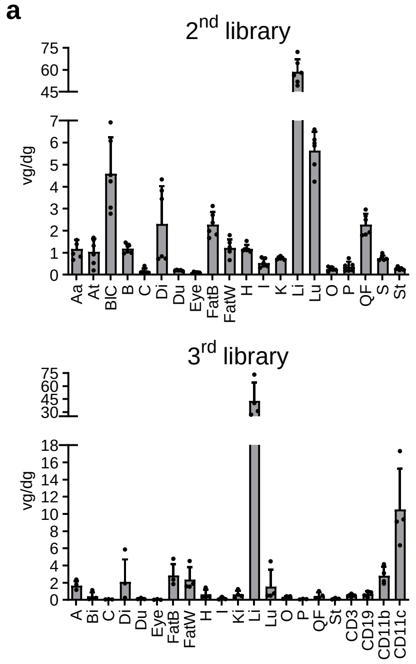


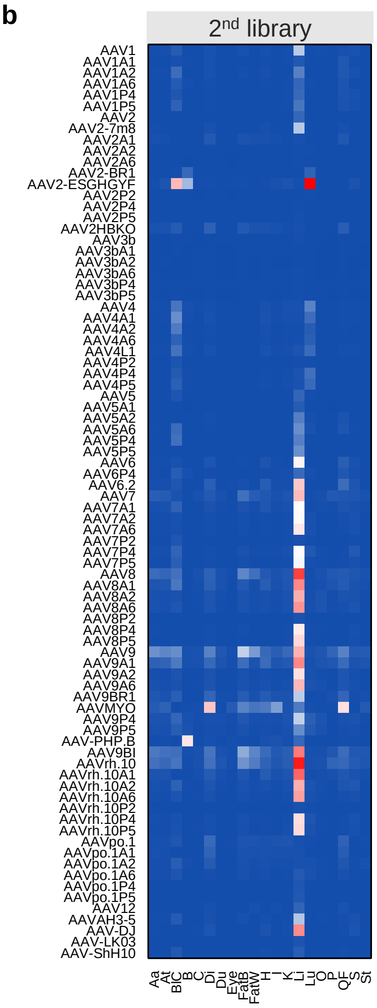


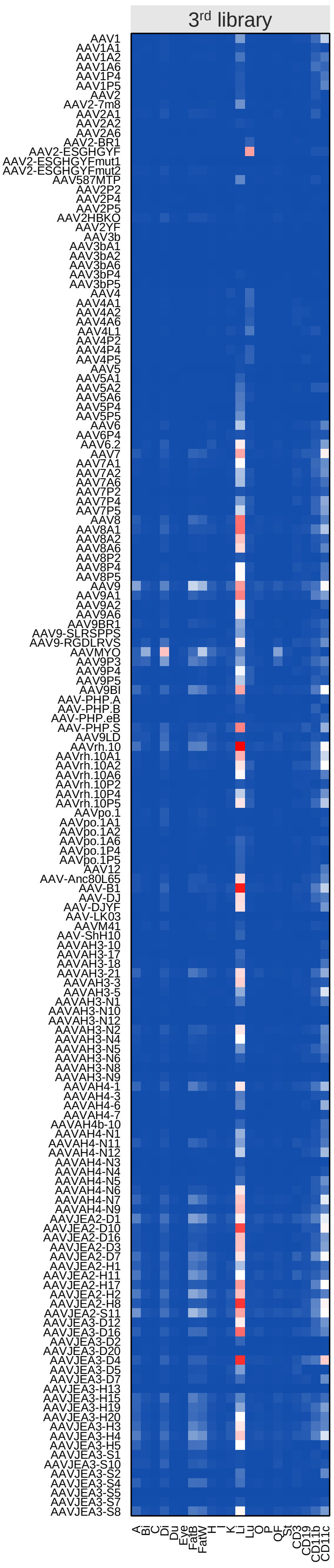


**Supplementary Fig. 2. Whole library and single variant biodistribution for 2nd and 3rd barcoded library.** (**a**) Viral DNA distribution of the 2nd and 3rd library after i.v. injection in aorta (A), abdominal aorta (Aa), thoracic aorta (At), brain (B), blood cells (BlC), biceps (Bi), colon (C), diaphragm (Di), duodenum (Du), eye, brown fat (FatB), white fat (FatW), heart (H), inner ear (I), kidney (K), liver (Li), lung (Lu), ovaries (O), pancreas (P), *quadriceps femoris* (QF), spleen (S), stomach (St) and CD11b-, CD11c-, CD19-, or CD3-positive cells. Detected viral genomes (EYFP probe) were normalized to GAPDH as a housekeeper. Depicted values represent the average viral genome per diploid genome (vg/dg) values from six (or four for 3rd library) C57BL/6J mice with SD. (**b**) Heat map simultaneously illustrating the transcriptional efficiency and specificity of all variants in the 2nd and 3rd library in aorta (A), abdominal aorta (Aa), thoracic aorta (At), brain (B), blood cells (BlC), biceps (Bi), colon (C), diaphragm (Di), duodenum (Du), eye, brown fat (FatB), white fat (FatW), heart (H), inner ear (I), kidney (K), liver (Li), lung (Lu), ovaries (O), pancreas (P), *quadriceps femoris* (QF), spleen (S), stomach (St) and CD11b-, CD11c-, CD19-, or CD3-positive cells. Depicted values represent the average $B_{\alpha\beta}$ values from six (or four for 3rd library) C57BL/6J mice with SD. A logarithmic scale is used with blue representing the value 0, white 0.47 and red 4.74 for the 2nd library and blue 0, white 0.37 and red 3.70 for the 3rd library.

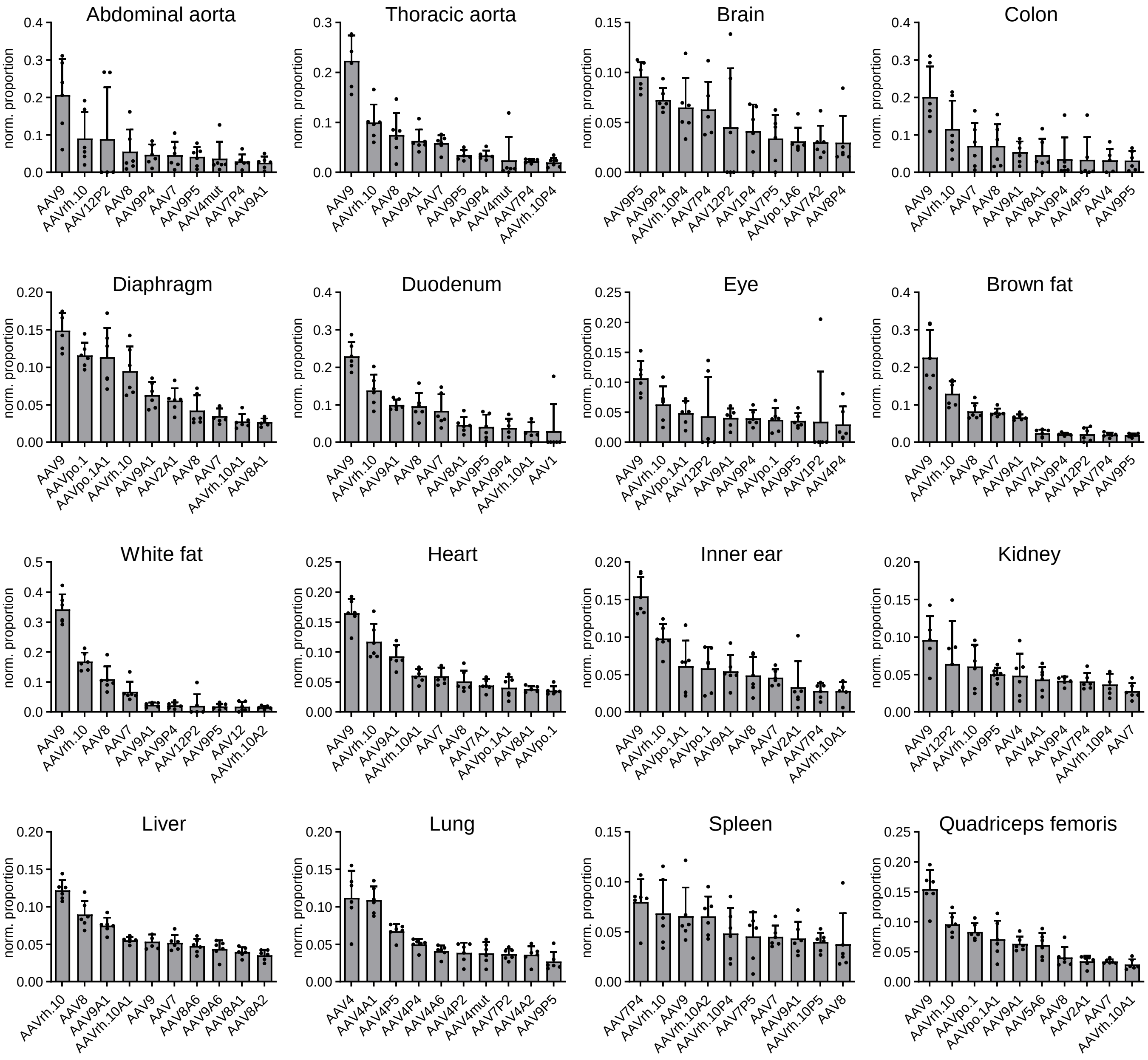


**Supplementary Fig. 3. Transcriptional efficiency in various tissues.** Top 10 AAV variants in the 1st barcoded library and the shown tissues based on normalized transcriptional efficiency ($V_{\alpha\beta}$). Depicted are average cDNA values from six C57BL/6J mice with SD.

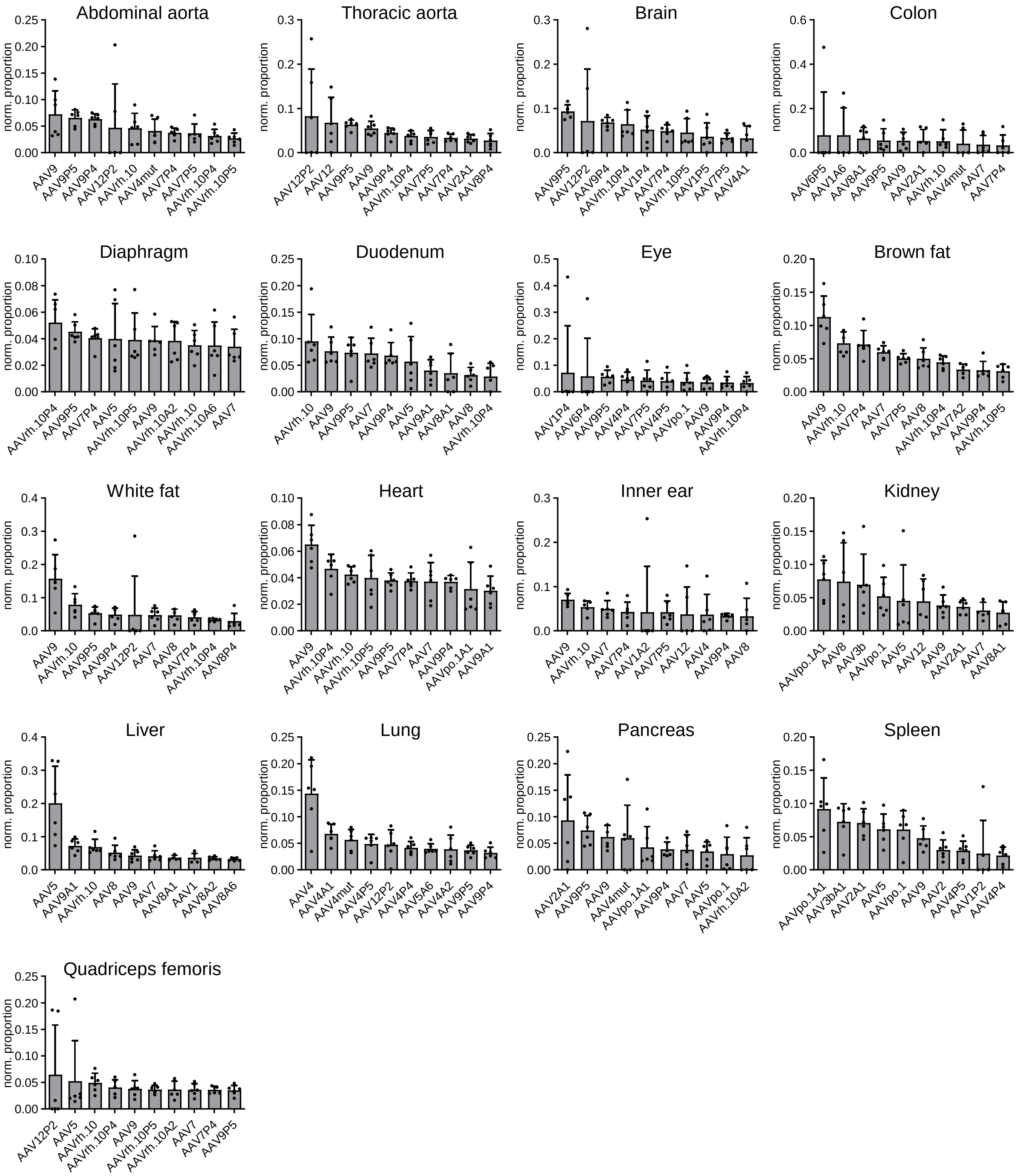


**Supplementary Fig. 4. Transduction efficiency in various tissues.** Top 10 AAV variants in the 1st barcoded library and the shown tissues based on normalized transduction efficiency ($V_{\alpha\beta}$). Depicted are average DNA values from six C57BL/6J mice with SD. Note that here and in all subsequent figures, "transduction efficiency" is defined by the capability of a given capsid to deliver detectable vector DNA to the target tissue.

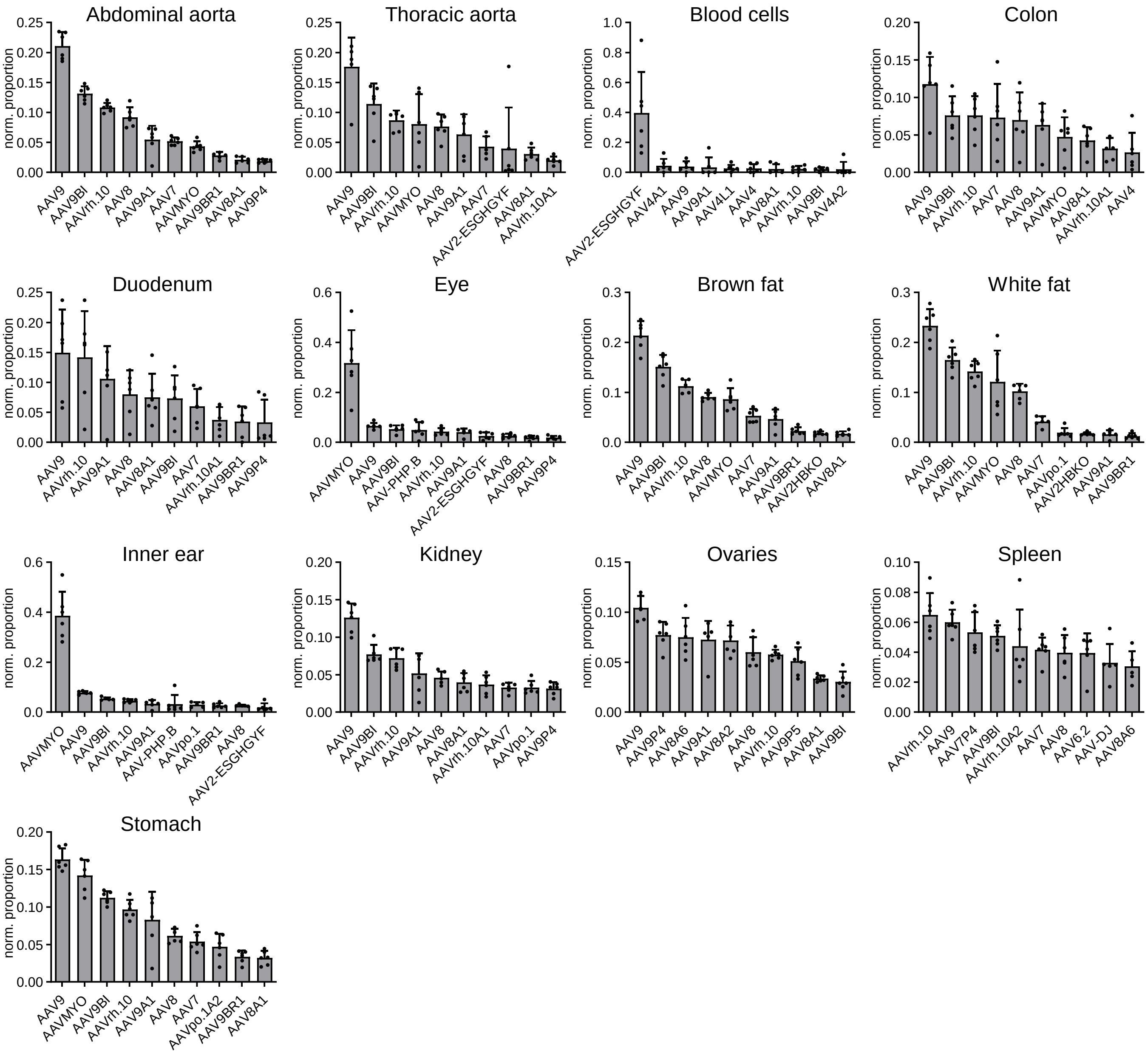


**Supplementary Fig. 5. Transcriptional efficiency in various tissues.** Top 10 AAV variants in the 2nd barcoded library and the shown tissues based on normalized transcriptional efficiency ($V_{\alpha\beta}$). Depicted are average cDNA values from six C57BL/6J mice with SD.

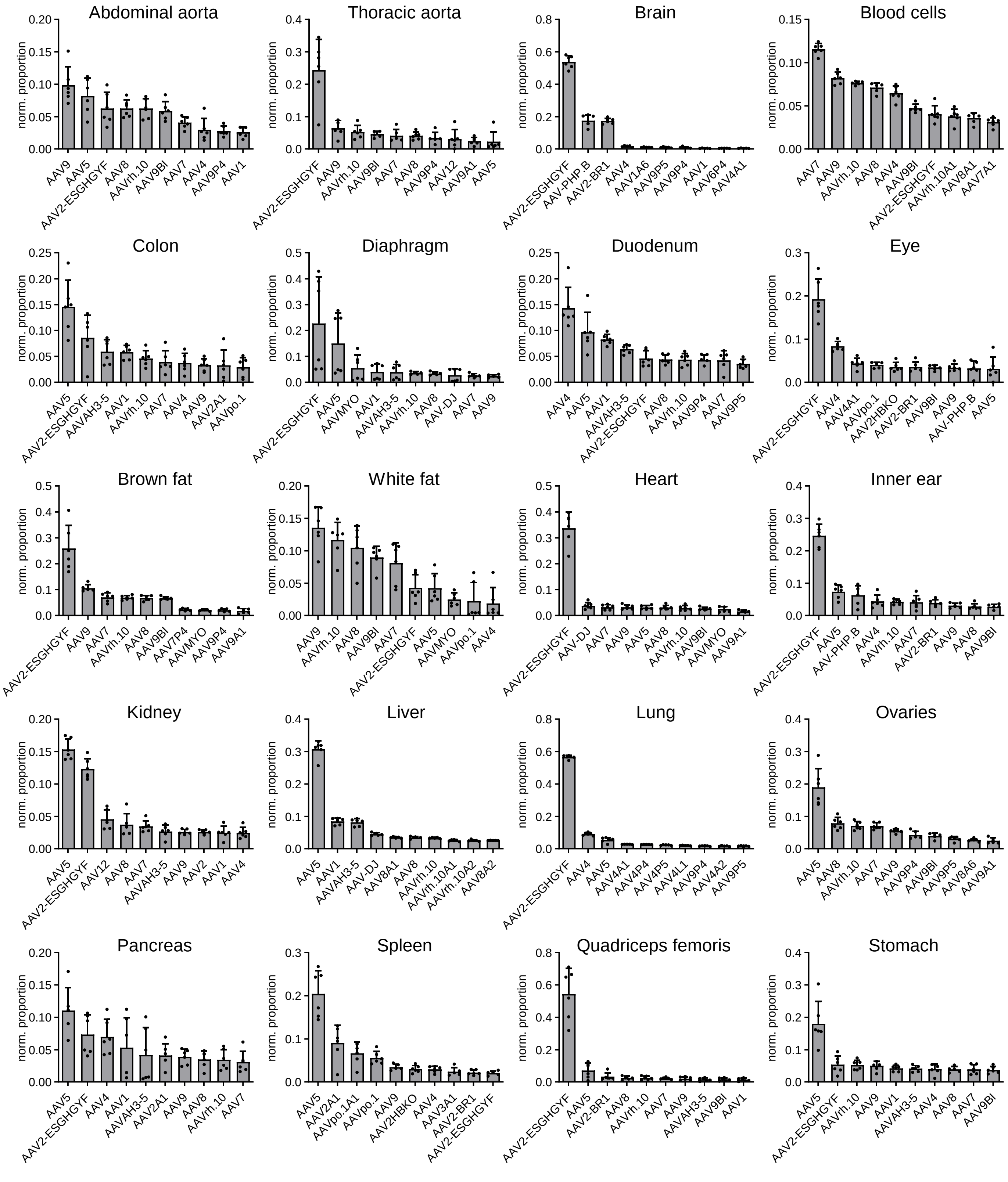


**Supplementary Fig. 6. Transduction efficiency in various tissues.** Top 10 AAV variants in the 2nd barcoded library and the shown tissues based on normalized transduction efficiency ($V_{\alpha\beta}$). Depicted are average DNA values from six C57BL/6J mice with SD.

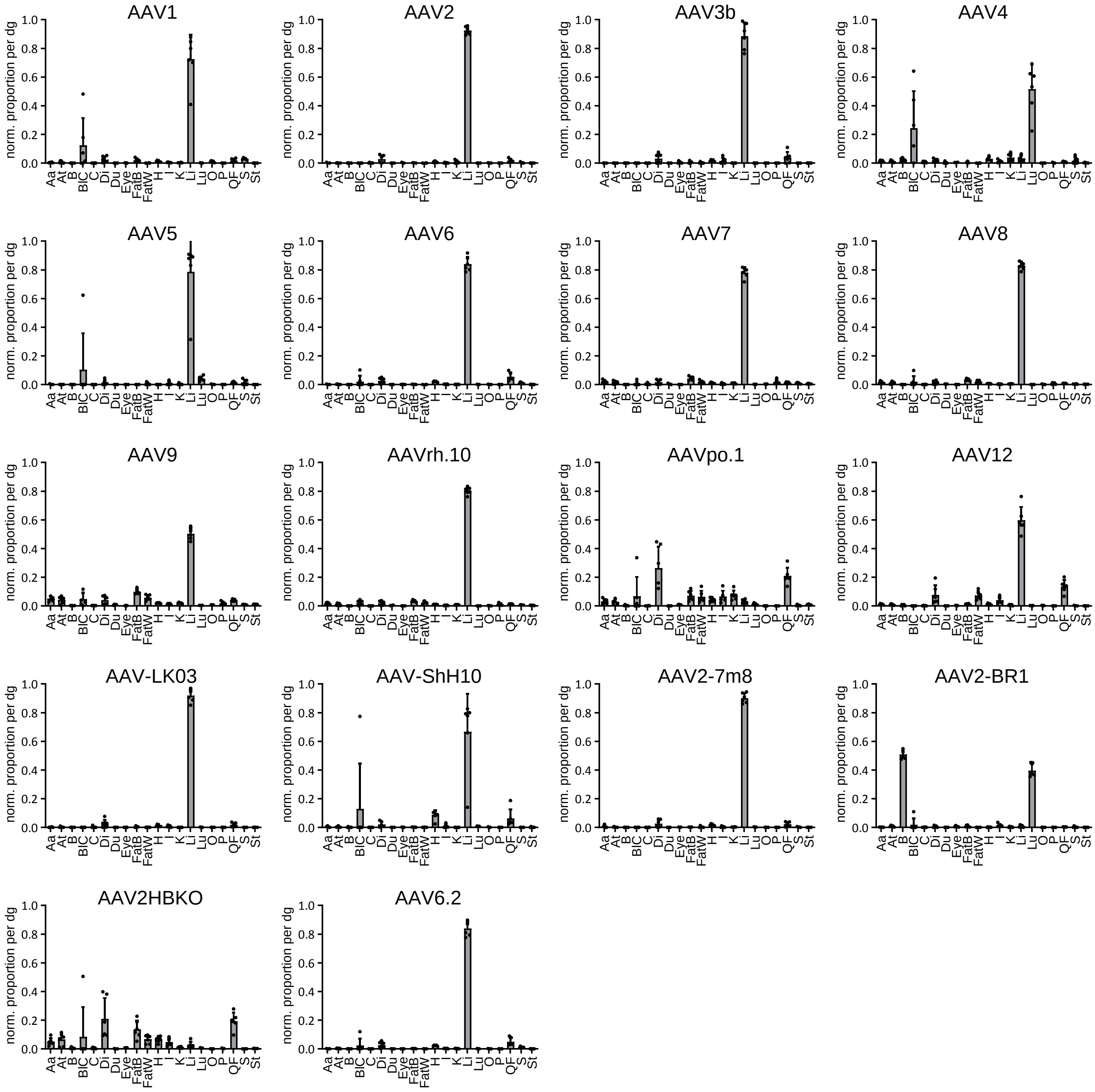


**Supplementary Fig. 7. Transcriptional specificity of common AAV serotypes and published variants.** Transcriptional specificity ($T_{\alpha\beta}$) of the shown capsids from the 2nd barcoded library as normalized proportion per cell (diploid genome, dg) in abdominal aorta (Aa), thoracic aorta (At), brain (B), blood cells (BlC), colon (C), diaphragm (Di), duodenum (Du), eye, brown fat (FatB), white fat (FatW), heart (H), inner ear (I), kidney (K), liver (Li), lung (Lu), ovaries (O), pancreas (P), *quadriceps femoris* (QF), spleen (S) and stomach (St). Depicted are average cDNA values from six C57BL/6J mice with SD.

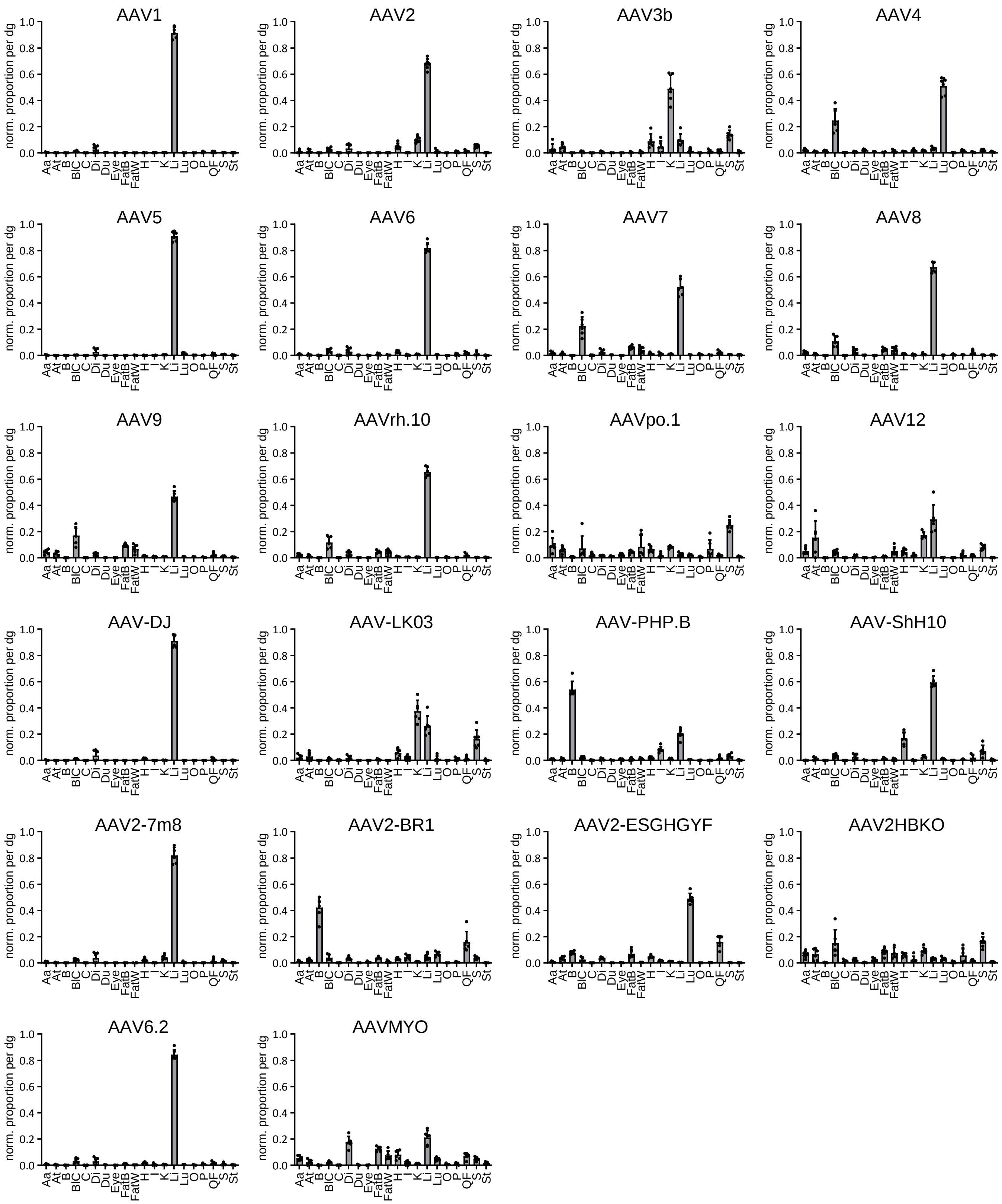


**Supplementary Fig. 8. Transduction specificity of common AAV serotypes, published variants and AAVMYO.** Transduction specificity ($T_{\alpha\beta}$) of the shown capsids from the 2nd barcoded library as normalized proportion per cell (diploid genome, dg) in abdominal aorta (Aa), thoracic aorta (At), brain (B), blood cells (BlC), colon (C), diaphragm (Di), duodenum (Du), eye, brown fat (FatB), white fat (FatW), heart (H), inner ear (I), kidney (K), liver (Li), lung (Lu), ovaries (O), pancreas (P), *quadriceps femoris* (QF), spleen (S) and stomach (St). Depicted are average DNA values from six C57BL/6J mice with SD.

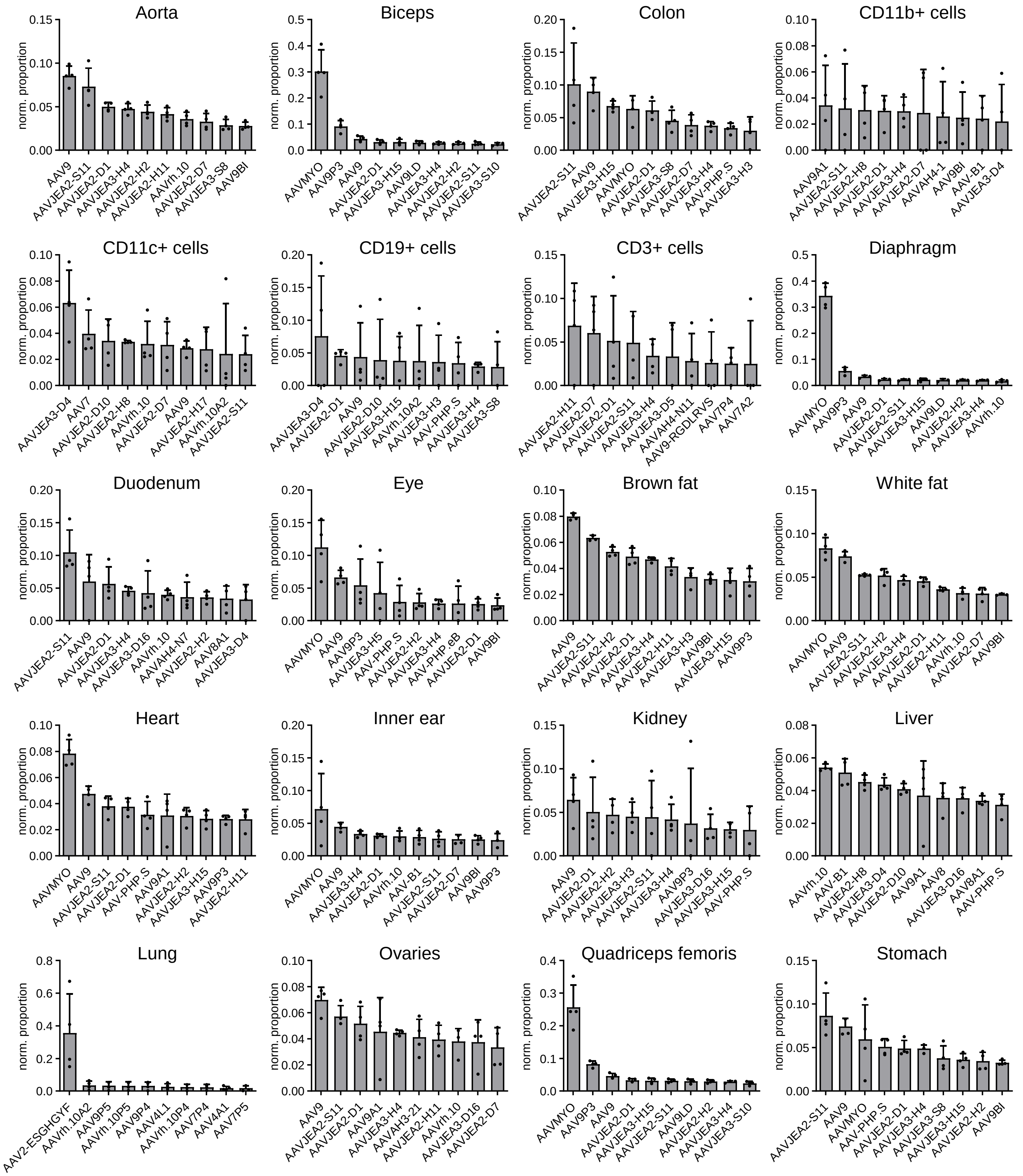


**Supplementary Fig. 9. Transcriptional efficiency in various tissues and cells.** Top 10 AAV variants in the 3rd barcoded library and the shown tissues based on normalized transcriptional efficiency ($V_{\alpha\beta}$). Depicted are average cDNA values from four C57BL/6J mice with SD.

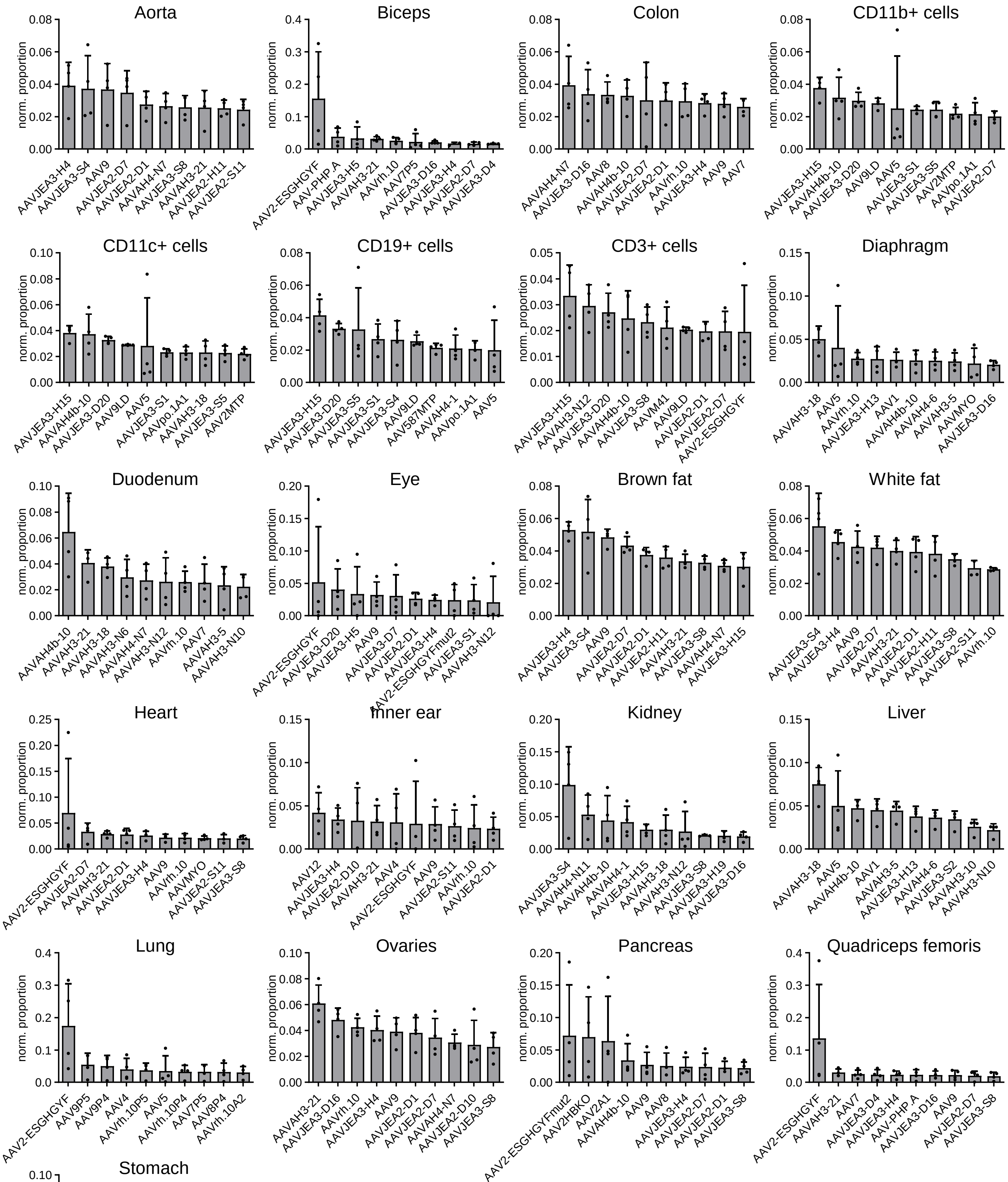


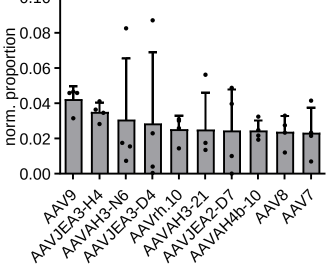


**Supplementary Fig. 10. Transduction efficiency in various tissues and cells.** Top 10 AAV variants in the 3rd barcoded library and the shown tissues based on normalized transduction efficiency ($V_{\alpha\beta}$). Depicted are average DNA values from four C57BL/6J mice with SD.

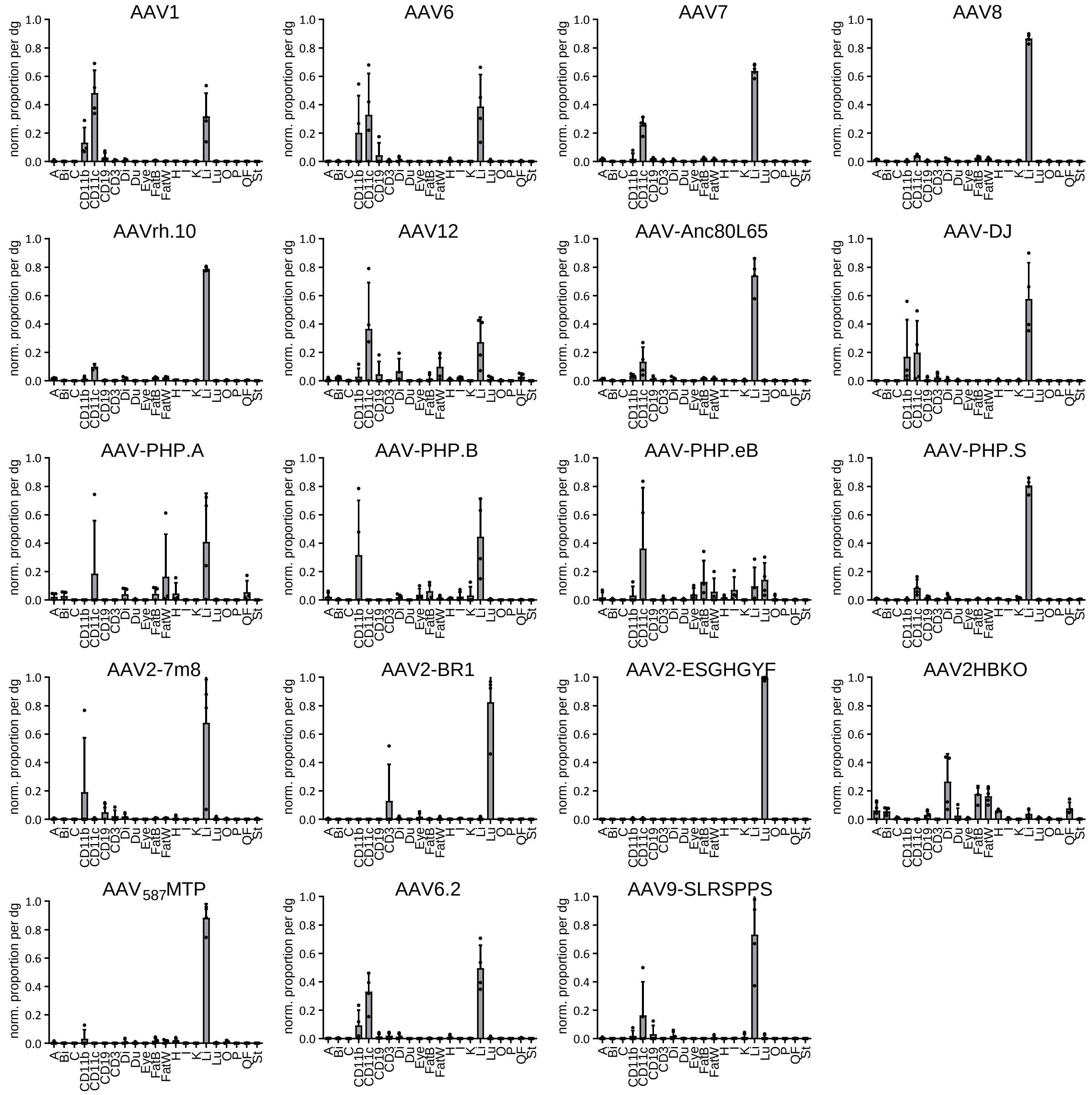


**Supplementary Fig. 11. Transcriptional specificity of common AAV serotypes and published variants.** Transcriptional specificity ($T_{\alpha\beta}$) of the shown capsids from the 3rd barcoded library as normalized proportion per cell (diploid genome, dg) in aorta (A), biceps (Bi), colon (C), CD11b-, CD11c-, CD19-, or CD3-positive cells, diaphragm (Di), duodenum (Du), eye, brown fat (FatB), white fat (FatW), heart (H), inner ear (I), kidney (K), liver (Li), lung (Lu), ovaries (O), pancreas (P), *quadriceps femoris* (QF) and stomach (St). Depicted are average cDNA values from four C57BL/6J mice with SD.

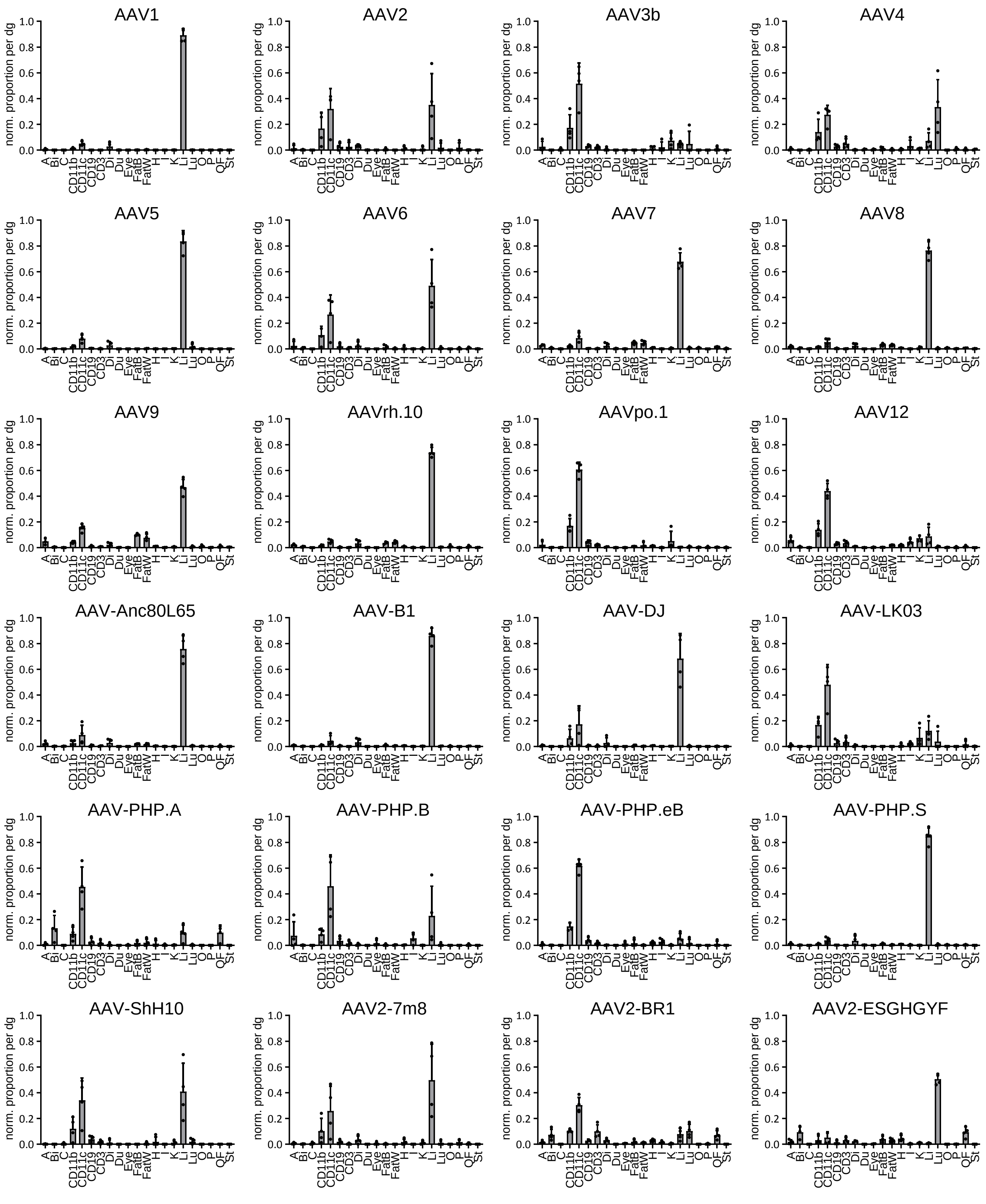


**Supplementary Fig. 12. Transduction specificity of common AAV serotypes and published variants.** Transduction specificity ($T_{\alpha\beta}$) of the shown capsids from the 3rd barcoded library as normalized proportion per cell (diploid genome, dg) in aorta (A), biceps (Bi), colon (C), CD11b-, CD11c-, CD19-, or CD3-positive cells, diaphragm (Di), duodenum (Du), eye, brown fat (FatB), white fat (FatW), heart (H), inner ear (I), kidney (K), liver (Li), lung (Lu), ovaries (O), pancreas (P), *quadriceps femoris* (QF) and stomach (St). Depicted are average DNA values from four C57BL/6J mice with SD.

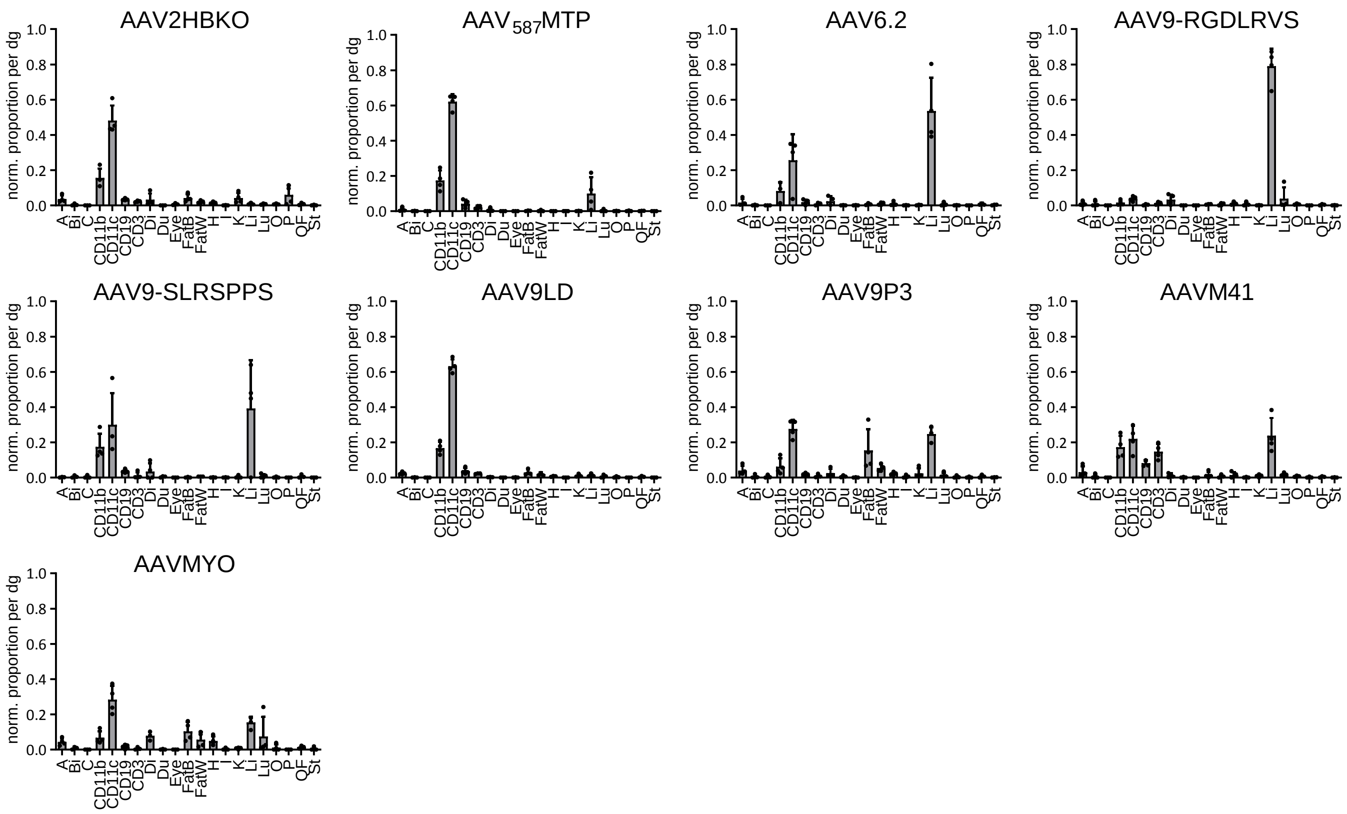


**Supplementary Fig. 13. Transduction specificity of published and novel AAV variants.** Transduction specificity ($T_{\alpha\beta}$) of the shown capsids from the 3rd barcoded library as normalized proportion per cell (diploid genome, dg) in aorta (A), biceps (Bi), colon (C), CD11b-, CD11c-, CD19-, or CD3-positive cells, diaphragm (Di), duodenum (Du), eye, brown fat (FatB), white fat (FatW), heart (H), inner ear (I), kidney (K), liver (Li), lung (Lu), ovaries (O), pancreas (P), *quadriceps femoris* (QF) and stomach (St). Depicted are average DNA values from four C57BL/6J mice with SD.

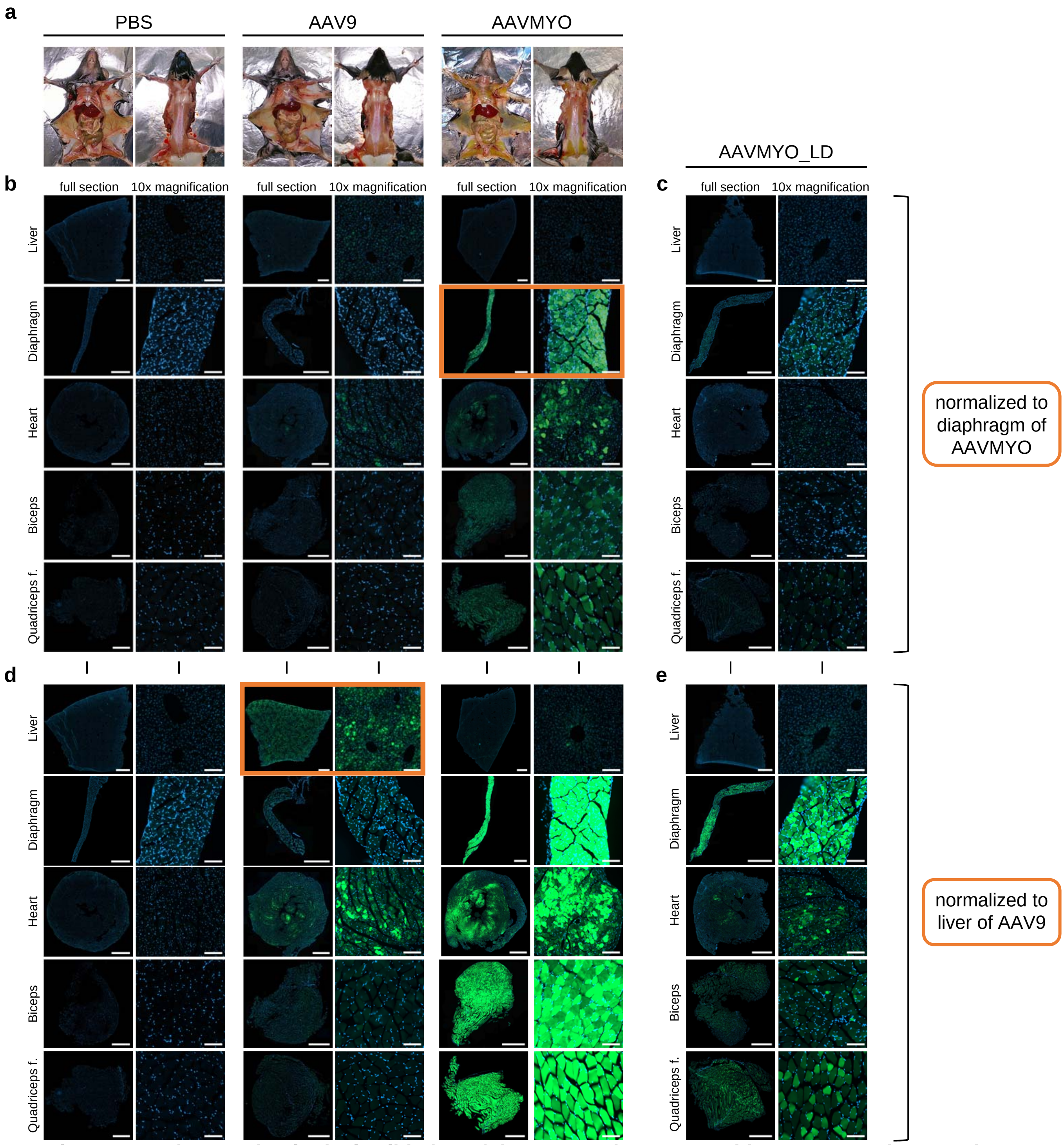


**Supplementary Fig. 14. Histological validation of the myotropic AAV capsid AAVMYO and comparison to a liver-detargeted variant**. (**a**) Representative images of dissected C57BL/6J mice (left: ventral; right: dorsal position) that were i.v. injected with 5×1011 vg per mouse and sacrificed two weeks later. Note: this panel is identical to Figure 3c and reproduced here for orientation. (**b**) Representative 10 µm cryosections (n = 8 replicates for AAV9, AAVMYO and n = 3 for PBS) of the liver, diaphragm, heart, biceps and *quadriceps femoris* of the mice from panel (a). Direct EGFP fluorescence was detected (green) together with the DAPI signal (blue). Exposure settings were normalized to the diaphragm of the AAVMYO group (highlighted by the orange box), which is different from Figure 3d where they were normalized to the liver of the AAV9 group. (**c**) Representative cryosections (n = 8 replicates) of a mouse injected with the AAVMYO_LD variant, using identical camera settings and scale bars as in panel (b). (**d**) Reproduction of Figure 3d, to allow for a direct comparison with the data in panel (e). Exposure settings were normalized to the liver of the AAV9 group (highlighted by the orange box). (**e**) Same samples as panel (c), but using the same camera settings as in panel (d). Scale bars for (b), (c), (d) and (e) are 1 mm (full sections) or 100 µm (10× magnifications).

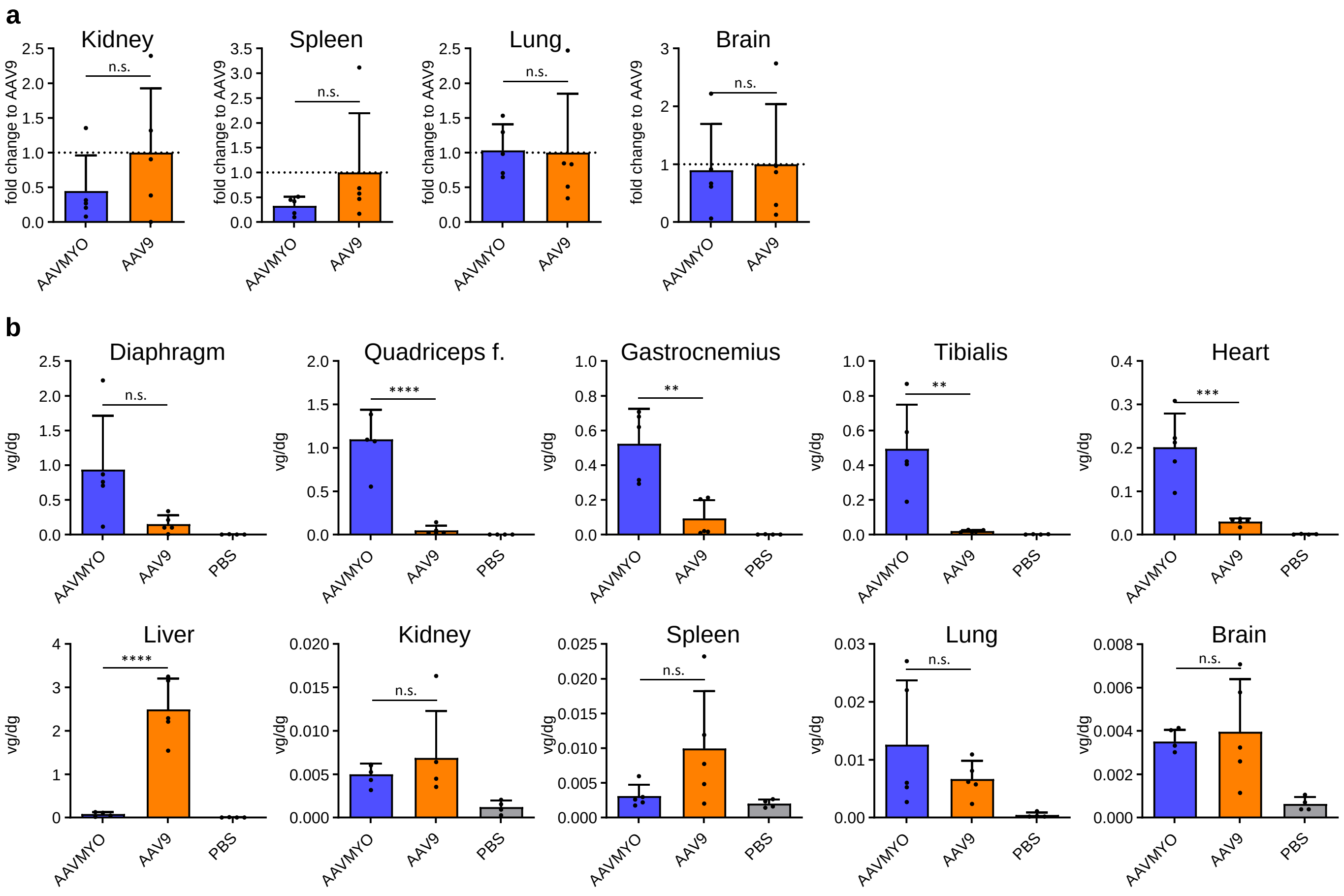


**Supplementary Fig. 15. Validation of AAVMYO by measurement of luciferase expression and vector genome biodistribution.** Shown are direct side-by-side comparisons of AAVMYO to AAV9 in individually i.v. injected mice. (**a**) Luciferase expression in different organs. AAVMYO values are depicted as fold-changes relative to AAV9 values (always set to 1). (**b**) Viral genomes per diploid genome (vg/dg) in the shown organs. Viral genomes were detected using a qPCR with Luc2-specific primers mentioned in the Methods section. Depicted values in (a) and (b) are the average of five CB17-SCID mice with SD. Colors in (a) and (b) highlight the same capsids in both panels. *p < 0.05, **p < 0.01, ***p < 0.001, ****p < 0.0001 (unpaired two-tailed t test for (a) and one-way ANOVA with Tukey's multiple comparison test for (b)).

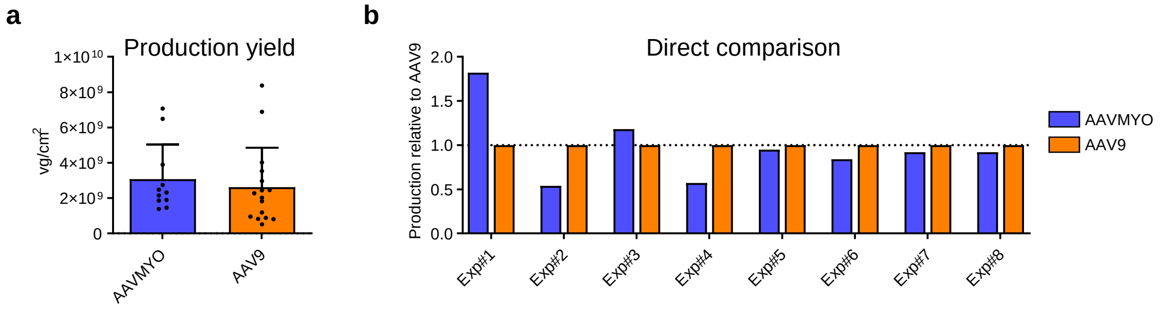


**Supplementary Fig. 16. AAVMYO vs AAV9 production efficiency comparison.** (**a**) Eleven independent productions of AAVMYO are compared with 16 productions of AAV9. Average total viral genomes (vg) per cm2 are depicted with SD. (**b**) Eight direct comparisons of AAVMYO and AAV9 were performed where both viruses were produced in the same experiment. Total vg/cm2 are shown relative to AAV9.

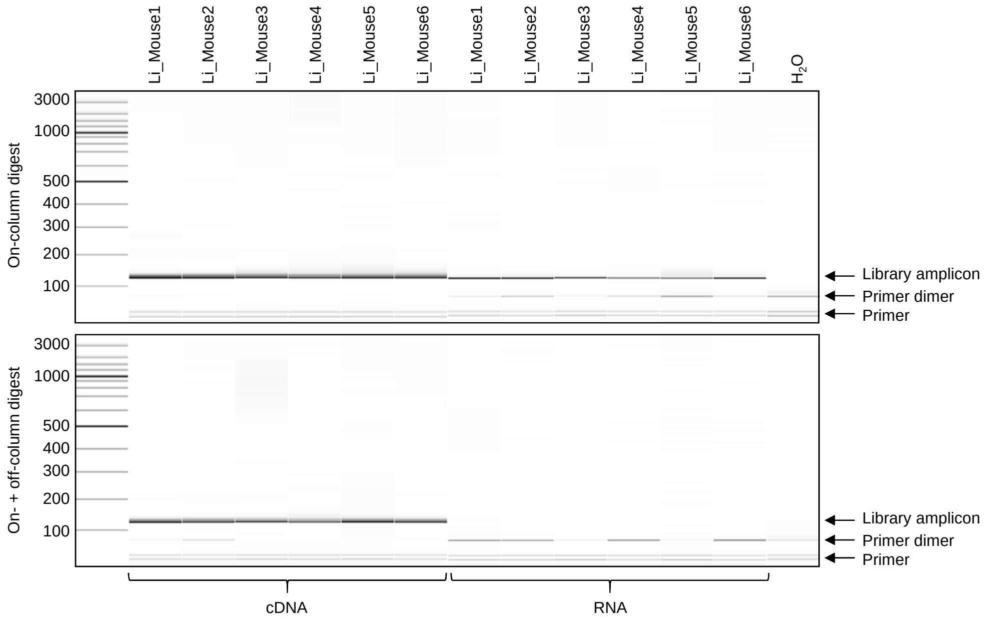


**Supplementary Fig. 17. DNase digest of isolated RNA.** Liver-extracted RNA of mouse 1-6 was used to establish PCR-based library amplification from resulting cDNA. The upper blot shows partial removal of contaminating gDNA by on-column DNase I digest, whereas the lower blot demonstrates complete removal of gDNA after additional off-column DNase I digest.

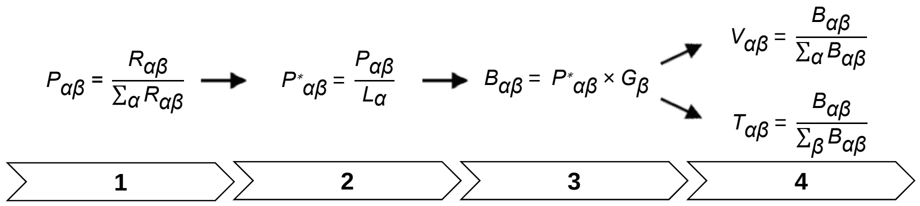


**Supplementary Fig. 18. NGS data normalization strategy.** Variant-assigned barcode read counts from Script#1_BarcodeDetection_v1.4 were used to normalize the data with Script#2_DataAnalysis_v2.24. The depicted workflow describes the calculations of Script#2. In the first step, the script normalizes the read counts $R$ of all variants $\alpha$ in tissue $\beta$ to the sum of all variants $\alpha$ in $\beta$ to obtain the proportion $P_{\alpha\beta}$. The second step normalizes $P_{\alpha\beta}$ to the proportion of each variant $\alpha$ in the initial library $L_\alpha$, which corrects for the uneven composition of the library. In the third step, $P^*_{\alpha\beta}$ is normalized to the qPCR-determined vg/dg, termed $G_\beta$, to allow for a comparison of one variant $\alpha$ over all analyzed tissues $\beta$. At this point, $B_{\alpha\beta}$ values were used and depicted directly to generate heat maps visualizing the differences of all variants $\alpha$ in all tissues $\beta$. $B_{\alpha\beta}$ values can also be shown as proportion of the sum over $\alpha$ or $\beta$ of $B_{\alpha\beta}$ (step 4). $V_{\alpha\beta}$ values were taken to create bar plots which demonstrate the proportion of all variants $\alpha$ in one tissue $\beta$ and therefore exemplify the efficiency of the individual vectors. Bar plots using $T_{\alpha\beta}$ values show the proportion of one variant $\alpha$ in all tissues $\beta$ allowing for an analysis of tissue specificity.

Supplementary Table 1. AAV capsid variants studied in this work.

| Variant | Source | 1st library | 2nd library | 3rd library |
|---|---|---|---|---|
| AAV1 | Herrmann[1] | x | x | x |
| AAV1A1 | Our lab | x | x | x |
| AAV1A2 | Our lab | x | x | x |
| AAV1A6 | Our lab | x | x | x |
| AAV1P2 | Our lab | x | | |
| AAV1P4 | Our lab | x | x | x |
| AAV1P5 | Our lab | x | x | x |
| AAV2 | Herrmann[1] | x | x | x |
| AAV2-7m8 | Dalkara[2] | | x | x |
| AAV2A1 | Our lab | x | x | x |
| AAV2A2 | Our lab | x | x | x |
| AAV2A6 | Our lab | x | x | x |
| AAV2-BR1 | Koerbelin[3] | | x | x |
| AAV2-ESGHGYF | Koerbelin[4] | | x | x |
| AAV2-ESGHGYFmut1 | Koerbelin[4] | | | x |
| AAV2-ESGHGYFmut2 | Koerbelin[4] | | | x |
| $AAV_{587}MTP$ | Yu[5] | | | x |
| AAV2P2 | Our lab | x | x | x |
| AAV2P4 | Our lab | x | x | x |
| AAV2P5 | Our lab | x | x | x |
| AAV2HBKO | Opie[6] | | x | x |
| AAV2YF | Li[7] | | | x |
| AAV3b | Herrmann[1] | x | x | x |
| AAV3bA1 | Our lab | x | x | x |
| AAV3bA2 | Our lab | x | x | x |
| AAV3bA6 | Our lab | x | x | x |
| AAV3bP2 | Our lab | x | | |
| AAV3bP4 | Our lab | x | x | x |
| AAV3bP5 | Our lab | x | x | x |
| AAV4 | Herrmann[1] | x | x | x |
| AAV4A1 | Our lab | x | x | x |
| AAV4A2 | Our lab | x | x | x |
| AAV4A6 | Our lab | x | x | x |
| AAV4L1 | Our lab | | x | x |
| AAV4P2 | Our lab | x | x | x |
| AAV4P4 | Our lab | x | x | x |
| AAV4P5 | Our lab | x | x | x |
| AAV4mut | Our lab | x | | |
| AAV4mutA1 | Our lab | x | | |
| AAV4mutA2 | Our lab | x | | |
| AAV4mutA6 | Our lab | x | | |
| AAV4mutP2 | Our lab | x | | |
| AAV4mutP4 | Our lab | x | | |
| AAV4mutP5 | Our lab | x | | |
| AAV5 | Herrmann[1] | x | x | x |
| AAV5A1 | Our lab | x | x | x |
| AAV5A2 | Our lab | x | x | x |
| AAV5A6 | Our lab | x | x | x |
| AAV5P2 | Our lab | x | | |
| AAV5P4 | Our lab | x | x | x |
| AAV5P5 | Our lab | x | x | x |
| AAV6 | Herrmann[1] | x | x | x |
| AAV6A1 | Our lab | x | | |
| AAV6A2 | Our lab | x | | |
| AAV6A6 | Our lab | x | | |
| AAV6P2 | Our lab | x | | |
| AAV6P4 | Our lab | x | x | x |
| AAV6P5 | Our lab | x | | |
| AAV6.2 | Limberis[8] | | x | x |
| AAV7 | Herrmann[1] | x | x | x |
| AAV7A1 | Our lab | x | x | x |
| AAV7A2 | Our lab | x | x | x |
| AAV7A6 | Our lab | x | x | x |
| AAV7P2 | Our lab | x | x | x |
| AAV7P4 | Our lab | x | x | x |
| AAV7P5 | Our lab | x | x | x |
| AAV8 | Herrmann[1] | x | x | x |
| AAV8A1 | Our lab | x | x | x |
| AAV8A2 | Our lab | x | x | x |
| AAV8A6 | Our lab | x | x | x |
| AAV8P2 | Our lab | x | x | x |
| AAV8P4 | Our lab | x | x | x |
| AAV8P5 | Our lab | x | x | x |
| AAV9 | Herrmann[1] | x | x | x |
| AAV9A1 | Our lab | x | x | x |
| AAV9A2 | Our lab | x | x | x |
| AAV9A6 | Our lab | x | x | x |

| Variant | Source | 1st library | 2nd library | 3rd library |
|---|---|---|---|---|
| AAV9BR1 | Our lab | | x | x |
| AAV9-SLRSPPS | Varadi[9] | | | x |
| AAV9-RGDLRVS | Varadi[9] | | | x |
| AAVMYO | Our lab | | x | x |
| AAV9P2 | Our lab | x | | |
| AAV9P3 | Our lab | | | x |
| AAV9P4 | Our lab | x | x | x |
| AAV9P5 | Our lab | x | x | x |
| AAV-PHP.A | Deverman[10] | | | x |
| AAV-PHP.B | Deverman[10] | | x | x |
| AAV-PHP.eB | Chan[11] | | | x |
| AAV-PHP.S | Chan[11] | | | x |
| AAV9BI | Our lab | | x | x |
| AAV9LD | Adachi[12] | | | x |
| AAVrh.10 | Herrmann[1] | x | x | x |
| AAVrh.10A1 | Our lab | x | x | x |
| AAVrh.10A2 | Our lab | x | x | x |
| AAVrh.10A6 | Our lab | x | x | x |
| AAVrh.10P2 | Our lab | x | x | x |
| AAVrh.10P4 | Our lab | x | x | x |
| AAVrh.10P5 | Our lab | x | x | x |
| AAVpo.1 | Herrmann[1] | x | x | x |
| AAVpo.1A1 | Our lab | x | x | x |
| AAVpo.1A2 | Our lab | x | x | x |
| AAVpo.1A6 | Our lab | x | x | x |
| AAVpo.1P2 | Our lab | x | | |
| AAVpo.1P4 | Our lab | x | x | x |
| AAVpo.1P5 | Our lab | x | x | x |
| AAV12 | Herrmann[1] | x | x | x |
| AAV12A1 | Our lab | x | | |
| AAV12A2 | Our lab | x | | |
| AAV12A6 | Our lab | x | | |
| AAV12P2 | Our lab | x | | |
| AAV12P4 | Our lab | x | | |
| AAV12P5 | Our lab | x | | |
| AAV-Anc80L65 | Zinn[13] | | | x |
| AAV-B1 | Choudhury[14] | | | x |
| AAV-DJ | Grimm[15] | | x | x |
| AAV-DJYF | Our lab | | | x |
| AAV-LK03 | Lisowski[16] | | x | x |
| AAVM41 | Yang[17] | | | x |
| AAV-ShH10 | Klimczak[18] | | x | x |
| AAVAH chimeras | Our lab | | | x |
| AAVJEA chimeras | Our lab | | | x |

Capsids are color-coded based on their origin: green = wild-type; yellow = peptide-displaying; purple = point mutants; turquoise = shuffled or ancestral.

Column "Source" lists references including the first author's last name. Note that the wild-type capsids have been published by numerous labs and that only one representative reference is given.

Supplementary Table 2. AAV barcodes used in this work.

| Plasmid name | Barcode sequence |
|---|---|
| pJW1-#A2 | AGACTCGTTGTATAT |
| pJW2-#A3 | TAGAGATTTAAACCG |
| pJW3-#A4 | CGTGACAGCGGATGG |
| pJW4-#A5 | TGGGCGGTCAGGGTC |
| pJW5-#A6 | TTGCCGTCCTTCGAG |
| pJW6-#A8 | TTCAGCGGACGGGCC |
| pJW7-#A9 | GTCAGTCCGCTCTTT |
| pJW8-#A11 | TTAAGATCCTGGTCG |
| pJW9-#A13 | TCAACATGGGCAACG |
| pJW10-#A14 | CTTGATCGACGCCCA |
| pJW11-#A15 | TACGCTATTCAATCT |
| pJW12-#A18 | GTGCTTCTGGCGGAT |
| pJW13-#A21 | CGGCTGTCGGTCGCC |
| pJW14-#A22 | ATCGTACGTTACTGA |
| pJW15-#A23 | GATTCGAAAGCATAG |
| pJW16-#A24 | CGTATCGGGTCCGGA |
| pJW17-#A25 | TGGTTGGGTTTGTGG |
| pJW18-#A26 | TCGTTGTAACGGTAC |
| pJW19-#A29 | TAACGTTGGGTTGCC |
| pJW20-#A30 | GACCACTAGAAGGGC |
| pJW21-#A32 | CTGCATGGCGGAGTT |
| pJW22-#A33 | TCAACGATTGTCTGG |
| pJW23-#A34 | TGGTAGGTTCGAAAT |
| pJW24-#A35 | ACGTCGCACCGTTTG |
| pJW25-#A37 | CAGGCTTAACGCGGG |
| pJW26-#A38 | ACCATAGCGCCACGA |
| pJW27-#A39 | GTCCCGACTAGGACT |
| pJW28-#A40 | GTCTTGATTGCTTCG |
| pJW29-#A41 | ATTTGGCACAGGATG |
| pJW30-#A42 | GGCCACCGTGTGTGA |
| pJW31-#A43 | ATGAGCAGCGAATGA |
| pJW32-#A44 | ATGTTTAACGGCATA |
| pJW33-#A45 | TTGGACTCACAGATG |
| pJW34-#A47 | AAGGTGACCTAGTGT |
| pJW35-#A48 | CCCTCATGAGGTCCG |
| pJW36-#A49 | ATGACAATGTGCAGG |
| pJW37-#A50 | GCGAGGTCGTTAGTT |
| pJW38-#A51 | TAAGACTGTTCCGGG |
| pJW39-#A52 | GTTTGTAATCTCTAC |
| pJW40-#A53 | GTTAACGCGGCCATT |
| pJW41-#A55 | AGCGGCGTTTATCGT |
| pJW42-#A56 | TTGGTATGTGTCAAT |
| pJW43-#A58 | GTCGACTTCATGGCA |
| pJW44-#A61 | GAGCGTAATTGTGAG |
| pJW45-#A62 | CGTTAACCCGAAAGC |
| pJW46-#A63 | GTGACATGCAGGTAG |
| pJW47-#A65 | ACGATCGTACGTCTT |
| pJW48-#A67 | GTTCAGGTCAGGTCT |
| pJW49-#A68 | TAAGGAGGGCTGTAG |
| pJW50-#A69 | TATCAAGCTAACGTT |
| pJW51-#A70 | GCTCTGGATGTAGTA |
| pJW52-#A71 | TAGATGTGGCGGACA |
| pJW53-#A74 | GTCAACATCGTTACA |
| pJW54-#A75 | GGGCCCTAGCGCGTG |
| pJW55-#A76 | GATAGGCTGGTCCAA |
| pJW56-#A77 | TATTTGTGTCGTTCC |
| pJW57-#A79 | AGTTAGGGCGCTGCG |
| pJW58-#A80 | GCGGAACATAGGCGG |
| pJW59-#A81 | GCCCTTCAGTCAGCT |
| pJW60-#A82 | CGGTCGCGTGACGTG |
| pJW61-#A83 | GCCGGAGTCCCGGTA |
| pJW62-#A84 | CGAGTCGTATGTGGC |
| pJW63-#A85 | AGTAATTGGTCTTGG |
| pJW64-#A86 | GGTCTTTGCTCGGTG |
| pJW65-#A87 | GACTTGGTTGTGACG |
| pJW66-#A90 | TTGTTGTATGAGCAG |
| pJW67-#A91 | TCCACGGAGGCTGCG |
| pJW68-#A94 | CTACCTATTTACTCT |
| pJW69-#A97 | ACCGGGCGTTGAGGC |
| pJW70-#A99 | ACTGTGATGGGTTAG |
| pJW71-#A100 | TGGTTTACAAATTAT |
| pJW72-#A101 | TGTCCGGAAAGGACA |
| pJW73-#A102 | GTTGTGCCCTGAGTG |
| pJW74-#A104 | ACCGTATCTCTCCGG |
| pJW75-#A107 | TTGGAACGTGGGCTT |
| pJW76-#A109 | AGATTCAAAGCTGCG |
| pJW77-#A110 | TGTTGGAAGGTATCA |
| pJW78-#A111 | GTAGCTGAGGTTGGT |
| pJW79-#A114 | AGCCTAATCTTTGAC |
| pJW80-#A115 | AAGCACTAAAGAACA |
| pJW81-#A116 | GGTATGGCCTGCCGC |
| pJW82-#A117 | TGTTTAGGTGAGCCT |
| pJW83-#A118 | TGTGGTGTGACTCAG |
| pJW84-#A119 | TCGGGTTGGTCTTTG |
| pJW85-#A120 | ACATTGTGGTCATAG |
| pJW86-#A121 | AGACTTGGCGTTATG |
| pJW87-#A122 | ACGTGTCGTAGTAAG |
| pJW88-#A124 | TATATTGAGGCGTGT |
| pJW89-#A126 | TGAGAGTCATCCAAG |
| pJW90-#A127 | CCTAATCTCAGGCGG |
| pJW91-#A129 | CGTGACCCAGGAAGT |
| pJW92-#A132 | TCGTTAGTAGCGATC |
| pJW93-#A135 | GAGGTCCAGAGGAAG |
| pJW94-#A138 | ATGATCAGCGATATC |
| pJW95-#A139 | GGTGCCGGACAGCTC |
| pJW96-#A141 | TATAACTTAGCTGAT |
| pJW97-#A142 | CTTCTTCAGGCAACC |
| pJW98-#A144 | CCACTAGGATCCGGA |
| pJW99-#A145 | CAAGGCTTTCTGATC |
| pJW100-#A146 | ATCTCGAAGCGCGTA |
| pJW101-#A147 | GCAATTATCATAGTC |
| pJW102-#A149 | GACCTGCGCCTTACA |
| pJW103-#A150 | CGTCCGTCTAATGAA |
| pJW104-#A151 | GGTTGACAGTGGGCT |
| pJW105-#A152 | AGTTTAGGACAGGCA |
| pJW106-#A155 | TTCATCGGCCGCTAA |
| pJW107-#A157 | TACGTATCGCGTGAT |
| pJW108-#A158 | CTAGGCAGGACACCG |
| pJW109-#A160 | TTGGCAGAGGATCAC |
| pJW110-#A161 | TCGGCTCTGTTCTAG |
| pJW111-#A162 | TTTAGGCGCGGCTTG |
| pJW112-#A163 | CGTCCTGTAAGGAGT |
| pJW113-#A164 | TAGAGTATGAGTGGT |
| pJW114-#A166 | GAGCGGGCAGACGAT |
| pJW115-#A169 | GTGCGCAGGTTAGTG |
| pJW116-#A171 | CTCGCGGCCTGAGGG |
| pJW117-#A172 | CTAGATAAATGCGGT |
| pJW118-#A173 | ACCTGAGTTTGGTGG |
| pJW119-#A175 | CCGTCGAAGAAGGGA |
| pJW120-#A179 | GGCAGCGGACACGTG |
| pJW121-#A180 | ATCCTCTCCGCTACC |
| pJW122-#A181 | TAGCACCATTTACGG |
| pJW123-#A184 | CATGCCATGTGTATC |
| pJW124-#A187 | ACCAACCGGTGTGGG |
| pJW125-#A189 | GGTACAGGACGCAGG |
| pJW126-#A190 | GACCACTTATCGCCA |
| pJW127-#A195 | TCGGCGTGGCGGTCG |
| pJW128-#A197 | GACTTTGACATGTCA |
| pJW129-#A198 | TACATTTAACTGAAG |
| pJW130-#A199 | GGTCAGGACCATTGG |
| pJW131-#A201 | TGGGTTTCGGCATCA |
| pJW132-#A202 | TTACCTTCTAAGGGC |
| pJW133-#A203 | TGGTCGGCGAGTTTG |
| pJW134-#A205 | GGTTGGTTAGGCTGT |
| pJW135-#A207 | ACCGGCAATCCTAGC |
| pJW136-#A208 | GTGTGTTACCTAACA |
| pJW137-#A209 | TCATCTAGCATCGGG |
| pJW138-#A210 | GCCACAGGCATCGTG |
| pJW139-#A211 | CTTATGTGAAGAGAT |
| pJW140-#A212 | TAGTTTATCGCAGGG |
| pJW141-#A213 | GTACCTATCCGTTGT |
| pJW142-#A214 | TTCCGTGTGTTGTCT |
| pJW143-#A215 | CCCGTATGTCGGGTA |
| pJW144-#A216 | GAATCCATGACTTTG |
| pJW145-#A217 | GTTCGTTGCGGGATC |
| pJW146-#A220 | GTGCTTGTCATGCCG |
| pJW147-#A221 | AGTTCACGACTGCGA |
| pJW148-#A222 | GGACTCAGGCCTGGT |
| pJW149-#A223 | TTTGGTTGGAGTCTT |
| pJW150-#A225 | TTACGATTTATGCGC |
| pJW151-#A226 | CAATCCGGCGCGGGT |
| pJW152-#A228 | GTGTAGGTTATCATC |
| pJW153-#A229 | TCGCACGCTGATGTG |
| pJW154-#A230 | AGTTTCACATGACGG |
| pJW155-#A232 | GTTTACGGATCTCGG |
| pJW156-#A233 | TATATAGTCGGTTTG |
| pJW157-#A236 | ATGTCGAACCCAATC |
| pJW158-#A237 | TCTGTATGGGCCAGC |
| pJW159-#A240 | TGATCTGACCGTGTG |

Supplementary Table 3. Primer sequences used in this work.

| **Primer name** | **Sequence 5’ to 3’** |
|---|---|
| Oligo_Barcode | TGACGTCTCTGCTCNNNNNNNNNNNNNNNCAGGCGAGACGTGACACTGC |
| 2ndStrand_Barcode_rev | GCAGTGTCACGTCTCGCCTG |
| NGS_Barcode_fw | ATCACTCTCGGCATGGACGAGC |
| NGS_Barcode_rev | GGCTGGCAACTAGAAGGCACA |
| qPCR_EYFP_fw | GAGCGCACCATCTTCTTCAAG |
| qPCR_EYFP_rev | TGTCGCCCTCGAACTTCAC |
| qPCR_Luc2_fw | CCCACCGTCGTATTCGTGAG |
| qPCR_Luc2_rev | TCAGGGCGATGGTTTTGTCCC |
| qPCR_mGAPDH_fw | TGTGTCCGTCGTGGATCTGA |
| qPCR_mGAPDH_rev | GCCTGCTTCACCACCTTCTTGA |

## SUPPLEMENTARY DISCUSSION

Our rationale in this study for inserting the barcodes into the 3' untranslated region of a CMV promoter-driven *eyfp* reporter gene was to enable tracking of all vector genomes on the DNA and RNA level, and to thus concurrently monitor and quantify the efficiency and specificity of the associated capsids. This, however, imposed challenges for the data normalization strategy.

Firstly, a comparison of barcoded viral transcripts across multiple organs assumes similar promoter activity in all analyzed tissue and cell types. We reckon that the CMV promoter may approximate this requirement and, thus, be a suitable candidate for our screenings, as it is ubiquitously expressed[19] and highly active within the short duration of the *in vivo* experiments performed here.

Secondly, to measure capsid specificity, the activity of each virus in every tissue had to be determined in order to calculate proportions in the individual organs (defined as specificity). While sequencing of the cDNA from each tissue reveals how much of all the detected barcode sequences belong to a given capsid variant, this proportion alone cannot be used to predict the specificity of this capsid across all tissues. For instance, a barcode/capsid could make up 20% of all variants in the liver and also 20% in the eye. However, these numbers solely describe the efficiency within the respective organ compared to all other variants in the library, yet they do not allow to conclude on the overall distribution of this capsid variant in the body. This fundamental difference - efficiency within a single tissue *versus* specificity across all studied tissues - is perhaps best illustrated by the bulk results in Supplementary Figure 2a, which show that most of each library ended up in the liver. Thus, even if a capsid has a high efficiency in a non-liver tissue as compared to all other capsids in the same tissue, its actual main target in the whole body may still be the liver itself, which was indeed frequently observed in this study.

Hence, it originally seemed ideal to determine the relative quantities of all viral transcripts in every organ and to then multiply this number with the barcode proportion obtained from the deep sequencing, resulting in the relative quantities of one variant in the corresponding organ. Yet,

this strategy would introduce a bias since the RNA expression levels of commonly used housekeeping genes vary across tissues[20, 21]. Therefore, the proportional values were normalized to the total viral genomes per cell in the cognate organ, by making the assumption that the genome delivered by a particular variant always produces the same amount of transcripts in all analyzed tissues. As mentioned above, this only applies if the promoter activity is identical in all tissues, which, however, cannot be guaranteed even for a ubiquitous promoter. Thus, determining specificity inevitably introduces a bias, either because of the heterogeneous housekeeper expression or because of the tissue-specific promoter activity.

Notably, assessing the relative quantities of the viral transcripts would require an additional qPCR step for every analyzed tissue, and the resulting values would moreover have to be divided by the total viral genomes ($G_β$) in the same organ. This calculation normalizes for the potentially unequal promoter activity but still cannot correct for the variable housekeeper expression.

In conclusion, both approaches are appropriate, yet the strategy that multiplies the NGS proportions solely with the $G_β$ values was chosen here as it saves one extra qPCR step.

Importantly, our normalization strategy also corrects for the unbalanced composition of the initial library and for total read count differences between flow cells, ultimately leading to so-called $B_{αβ}$ values (see Methods) that describe the overall biodistribution of every vector in the screening. These values can be depicted as proportion of one variant across all tissues ($T_{αβ}$ values) or as proportion of all variants within one tissue ($V_{αβ}$ values). The same formulas were also used to process the DNA dataset. Of note, as the viral genomes—unlike the transcripts—are independent of the CMV promoter activity, this analysis was unbiased.

In summary, by permitting to simultaneously monitor capsid specificity and efficiency, the normalization procedure used here enables the comprehensive, sensitive and high-throughput stratification of pre-selected AAV vectors, and thereby closes a pivotal gap in their development as clinical gene therapeutics.

## REFERENCES


1. Herrmann, A.K. et al. A Robust and All-Inclusive Pipeline for Shuffling of Adeno-Associated Viruses. *ACS Synth. Biol.* **8,** 194-206 (2019).
2. Dalkara, D. et al. In vivo-directed evolution of a new adeno-associated virus for therapeutic outer retinal gene delivery from the vitreous. *Sci. Transl. Med.* **5,** 189ra176 (2013).
3. Korbelin, J. et al. A brain microvasculature endothelial cell-specific viral vector with the potential to treat neurovascular and neurological diseases. *EMBO Mol. Med.* **8,** 609-625 (2016).
4. Korbelin, J. et al. Pulmonary Targeting of Adeno-associated Viral Vectors by Next-generation Sequencing-guided Screening of Random Capsid Displayed Peptide Libraries. *Mol. Ther.* **24,** 1050-1061 (2016).
5. Yu, C.Y. et al. A muscle-targeting peptide displayed on AAV2 improves muscle tropism on systemic delivery. *Gene Ther.* **16,** 953-962 (2009).
6. Opie, S.R., Warrington, K.H., Jr., Agbandje-McKenna, M., Zolotukhin, S. & Muzyczka, N. Identification of amino acid residues in the capsid proteins of adeno-associated virus type 2 that contribute to heparan sulfate proteoglycan binding. *J. Virol.* **77,** 6995-7006 (2003).
7. Li, M. et al. High-efficiency transduction of fibroblasts and mesenchymal stem cells by tyrosine-mutant AAV2 vectors for their potential use in cellular therapy. *Hum. Gene Ther.* **21,** 1527-1543 (2010).
8. Limberis, M.P., Vandenberghe, L.H., Zhang, L., Pickles, R.J. & Wilson, J.M. Transduction efficiencies of novel AAV vectors in mouse airway epithelium in vivo and human ciliated airway epithelium in vitro. *Mol. Ther.* **17,** 294-301 (2009).
9. Varadi, K. et al. Novel random peptide libraries displayed on AAV serotype 9 for selection of endothelial cell-directed gene transfer vectors. *Gene Ther.* **19,** 800-809 (2012).

10. Deverman, B.E. et al. Cre-dependent selection yields AAV variants for widespread gene transfer to the adult brain. *Nat. Biotechnol.* **34,** 204-209 (2016).

11. Chan, K.Y. et al. Engineered AAVs for efficient noninvasive gene delivery to the central and peripheral nervous systems. *Nat. Neurosci.* **20,** 1172-1179 (2017).

12. Adachi, K., Enoki, T., Kawano, Y., Veraz, M. & Nakai, H. Drawing a high-resolution functional map of adeno-associated virus capsid by massively parallel sequencing. *Nat. Commun.* **5,** 3075 (2014).

13. Zinn, E. et al. In Silico Reconstruction of the Viral Evolutionary Lineage Yields a Potent Gene Therapy Vector. *Cell Rep.* **12,** 1056-1068 (2015).

14. Choudhury, S.R. et al. In Vivo Selection Yields AAV-B1 Capsid for Central Nervous System and Muscle Gene Therapy. *Mol. Ther.* **24,** 1247-1257 (2016).

15. Grimm, D. et al. In vitro and in vivo gene therapy vector evolution via multispecies interbreeding and retargeting of adeno-associated viruses. *J. Virol.* **82,** 5887-5911 (2008).

16. Lisowski, L. et al. Selection and evaluation of clinically relevant AAV variants in a xenograft liver model. *Nature* **506,** 382-386 (2014).

17. Yang, L. et al. A myocardium tropic adeno-associated virus (AAV) evolved by DNA shuffling and in vivo selection. *Proc. Natl. Acad. Sci. U S A* **106,** 3946-3951 (2009).

18. Klimczak, R.R., Koerber, J.T., Dalkara, D., Flannery, J.G. & Schaffer, D.V. A novel adeno-associated viral variant for efficient and selective intravitreal transduction of rat Muller cells. *PLoS One* **4,** e7467 (2009).

19. Schmidt, E.V., Christoph, G., Zeller, R. & Leder, P. The cytomegalovirus enhancer: a pan-active control element in transgenic mice. *Mol. Cell. Biol.* **10,** 4406-4411 (1990).

20. Kouadjo, K.E., Nishida, Y., Cadrin-Girard, J.F., Yoshioka, M. & St-Amand, J. Housekeeping and tissue-specific genes in mouse tissues. *BMC Genomics* **8,** 127 (2007).

21. Zeng, J. et al. Identification and analysis of house-keeping and tissue-specific genes based on RNA-seq data sets across 15 mouse tissues. *Gene* **576,** 560-570 (2016).
