# Supplementary material for: Identification of a myotropic AAV by massively parallel in vivo evaluation of barcoded capsid variants
Source: Nat Commun. 2020 Oct 28;11:5432. doi: 10.1038/s41467-020-19230-w (PMC7595228; doi:10.1038/s41467-020-19230-w)
Supplement: Supplementary file 2 — Reporting Summary [file 41467_2020_19230_MOESM2_ESM.pdf]

nature research

Corresponding author(s): Dirk Grimm

Last updated by author(s): Sep 14, 2020

Reporting Summary

Nature Research wishes to improve the reproducibility of the work that we publish. This form provides structure for consistency and transparency in reporting. For further information on Nature Research policies, see our Editorial Policies and the Editorial Policy Checklist.

Statistics

For all statistical analyses, confirm that the following items are present in the figure legend, table legend, main text, or Methods section.

n/a	Confirmed	
☐	☒	The exact sample size (*n*) for each experimental group/condition, given as a discrete number and unit of measurement
☐	☒	A statement on whether measurements were taken from distinct samples or whether the same sample was measured repeatedly
☐	☒	The statistical test(s) used AND whether they are one- or two-sided<br>*Only common tests should be described solely by name; describe more complex techniques in the Methods section.*
☒	☐	A description of all covariates tested
☒	☐	A description of any assumptions or corrections, such as tests of normality and adjustment for multiple comparisons
☐	☒	A full description of the statistical parameters including central tendency (e.g. means) or other basic estimates (e.g. regression coefficient) AND variation (e.g. standard deviation) or associated estimates of uncertainty (e.g. confidence intervals)
☐	☒	For null hypothesis testing, the test statistic (e.g. *F*, *t*, *r*) with confidence intervals, effect sizes, degrees of freedom and *P* value noted<br>*Give P values as exact values whenever suitable.*
☒	☐	For Bayesian analysis, information on the choice of priors and Markov chain Monte Carlo settings
☒	☐	For hierarchical and complex designs, identification of the appropriate level for tests and full reporting of outcomes
☒	☐	Estimates of effect sizes (e.g. Cohen's *d*, Pearson's *r*), indicating how they were calculated

Our web collection on statistics for biologists contains articles on many of the points above.

Software and code

Policy information about availability of computer code

Data collection	Python 2.7 scripts were used for data collection and analysis. The data collection script was uploaded to GitHub: https://github.com/JonasWeinmann/AAV-barcode-detection-and-normalization
Data analysis	Python 2.7 scripts were used for data collection and analysis. The data analysis script was uploaded to GitHub: https://github.com/JonasWeinmann/AAV-barcode-detection-and-normalization. M3Vision software was used to analyze raw images from mouse organs containing raw data. GraphPad Prism 8 was used for statistical analyses.

For manuscripts utilizing custom algorithms or software that are central to the research but not yet described in published literature, software must be made available to editors and reviewers. We strongly encourage code deposition in a community repository (e.g. GitHub). See the Nature Research guidelines for submitting code & software for further information.

Data

Policy information about availability of data

All manuscripts must include a data availability statement. This statement should provide the following information, where applicable:

- Accession codes, unique identifiers, or web links for publicly available datasets
- A list of figures that have associated raw data
- A description of any restrictions on data availability

All data generated or analyzed during this study are included in this published article and its Supplementary information files. Raw sequencing data are available via accession code PRJNA557319 [https://www.ncbi.nlm.nih.gov/Traces/study/?acc=PRJNA557319]. The complete nucleotide sequence of AAVMYO can be found under GenBank accession code MN365014. Alternatively, its sequence as well as the complete sequence of the AAVMYO helper plasmid can be directly obtained from the authors upon request. Source data are provided with this paper.

Field-specific reporting

Please select the one below that is the best fit for your research. If you are not sure, read the appropriate sections before making your selection.

- [x] Life sciences
- [ ] Behavioural & social sciences
- [ ] Ecological, evolutionary & environmental sciences

For a reference copy of the document with all sections, see nature.com/documents/nr-reporting-summary-flat.pdf

Life sciences study design

All studies must disclose on these points even when the disclosure is negative.

Sample size	Sample size was chosen depending on the technique used and based on our experience. No power calculations were performed to choose group size. At least 3 animals were used per group. In the case of the in vivo capsid library screens, this was based on experience in our lab with the minimum number of animals needed to obtain sufficient amounts of DNA and RNA from all tissues and cell types of interest for subsequent next-generation sequencing analysis.
Data exclusions	All samples were included in the analyses. No data was excluded.
Replication	Every experiment was repeated and reproduced at least two times, and where appropriate the representative result is presented. The number of independent experiments and the number of biological replicates are stated for each chart.
Randomization	This study exclusively uses mice as experimental animals. For each cohort within the various experiments, inbred mice were randomly selected from a group of mice of the same age and sex plus similar weight to standardize the control and experimental groups as much as possible.
Blinding	Investigators were not blinded to group allocations because all in vivo experiments were conducted by the same person. Moreover, the in vivo library screens always comprised a single group, i.e., the AAV library, hence blinding was not required.

Reporting for specific materials, systems and methods

We require information from authors about some types of materials, experimental systems and methods used in many studies. Here, indicate whether each material, system or method listed is relevant to your study. If you are not sure if a list item applies to your research, read the appropriate section before selecting a response.

Materials & experimental systems

n/a	Involved in the study	
[ ]	[x]	Antibodies
[ ]	[x]	Eukaryotic cell lines
[x]	[ ]	Palaeontology and archaeology
[ ]	[x]	Animals and other organisms
[x]	[ ]	Human research participants
[x]	[ ]	Clinical data
[x]	[ ]	Dual use research of concern

Methods

n/a	Involved in the study	
[x]	[ ]	ChIP-seq
[x]	[ ]	Flow cytometry
[x]	[ ]	MRI-based neuroimaging

Antibodies

Antibodies used	1) Rabbit polyclonal anti-dystrophin antibody (Thermo Fisher Scientific, Cat#RB-9024-P, diluted 1:500)<br>2) Alexa Fluor 546 donkey polyclonal anti-rabbit secondary antibody (Thermo Fisher Scientific, Cat# A10040, diluted 1:400)<br>3) Mouse monoclonal anti-GAPDH antibody (Merck, Cat#G8795, diluted 1:5.000)<br>4) Donkey Anti-rabbit IgG-HRP secondary antibody (Santa Cruz, Cat#sc-2305, diluted 1:10.000)<br>5) Goat Anti-mouse IgG-HRP secondary antibody (Santa Cruz, Cat#sc-2302, diluted 1:10.000)<br>6) Rabbit Alexa 488 polyclonal anti-GFP antibody (Thermo Fischer Scientific, Cat#A-21311)<br>7) Bovine monoclonal anti-myosin heavy chain type I antibody (Developmental Studies Hybridoma Bank, Cat#BA-F8, diluted 1:50)<br>8) Bovine monoclonal anti-myosin heavy chain type IIA antibody (Developmental Studies Hybridoma Bank, Cat#sc-71, diluted 1:100)<br>9) Bovine monoclonal anti-myosin heavy chain type IIB antibody (Developmental Studies Hybridoma Bank, Cat#BF-F3, diluted 1:500)<br>10) Alexa Fluor 546 goat polyclonal anti-mouse IgM antibody (Thermo Fisher Scientific, Cat#A-21045, diluted 1:400)<br>11) Alexa Fluor 647 goat polyclonal anti-mouse IgG2b antibody (Thermo Fisher Scientific, Cat#A-21242, diluted 1:400)<br>12) Alexa Fluor 647 goat polyclonal anti-mouse IgG1 antibody (Thermo Fisher Scientific, Cat#A-21240, diluted 1:400)
Validation	All commercially available antibodies are commonly used and were validated by the suppliers, as documented below.<br><br>1) https://www.thermofisher.com/order/catalog/product/RB-9024-P#/RB-9024-P<br>2) https://www.thermofisher.com/antibody/product/Donkey-anti-Rabbit-IgG-H-L-Highly-Cross-Adsorbed-Secondary-Antibody-

Polyclonal/A10040
3) https://www.sigmaaldrich.com/catalog/product/sigma/g8795?lang=en&region=US
4) https://datasheets.scbt.com/sc-2305.pdf
5) https://datasheets.scbt.com/sc-2302.pdf
6) https://www.thermofisher.com/antibody/product/GFP-Antibody-Polyclonal/A-21311
7) https://dshb.biology.uiowa.edu/BA-F8
8) https://dshb.biology.uiowa.edu/SC-71
9) https://dshb.biology.uiowa.edu/BF-F3
10) https://www.thermofisher.com/antibody/product/Goat-anti-Mouse-IgM-Heavy-chain-Cross-Adsorbed-Secondary-Antibody-Polyclonal/A-21045
11) https://www.thermofisher.com/antibody/product/Goat-anti-Mouse-IgG2b-Cross-Adsorbed-Secondary-Antibody-Polyclonal/A-21242
12) https://www.thermofisher.com/antibody/product/Goat-anti-Mouse-IgG1-Cross-Adsorbed-Secondary-Antibody-Polyclonal/A-21240

Eukaryotic cell lines

Policy information about cell lines

Cell line source(s)
AAV-293 (HEK-293) (not listed in ICLAC register; see below). This cell line is commercially available from Stratagene/Agilent (http://www.integratedsci.com.au/product/aav-293-cells.html Catalog Code: 240073)

Authentication
The cell line was not authenticated because it was obtained commercially.

Mycoplasma contamination
The cell line was tested negative for mycoplasma contamination.

Commonly misidentified lines (See ICLAC register)
No commonly misidentified cell lines were used in the study.

Animals and other organisms

Policy information about studies involving animals; ARRIVE guidelines recommended for reporting animal research

Laboratory animals
Seven-weeks-old female inbred C57BL/6J mice (Janvier Labs) were used for all in vivo library screens as well as for the AAVMYO validation experiments.
Native gfp staining in the muscle was conducted in six-weeks-old female C57BL/6 mice (Fig. 3d). Muscle fiber staining was conducted in eight-weeks-old male C57BL/6 mice (Fig. 4d).
All in vivo and ex vivo luciferase imaging procedures were conducted in four-weeks-old male CB17-SCID mice.
Analysis of µDys expression was performed in six-weeks-old male mdx mice (Fig. 4c).
All animals were housed at approximately 21 degrees celsius at 50-60% humidity. All mice were housed in the animal facility at Heidelberg or Brussels. SCID mice were housed in IVC systems.

Wild animals
The study did not involve wild animals.

Field-collected samples
The study did not involve field-collected samples.

Ethics oversight
Library screens and most AAVMYO validation experiments were performed based on animal protocols 35-9185.81/G-126/14 and 35-9185.81/G-89/16 that were approved by the Regierungspräsidium Karlsruhe (Germany).
Luciferase in vivo studies were approved by the institutional animal ethics committee of the Free University of Brussels (VUB) (Brussels, Belgium).
For the dystrophin experiments, all procedures involving the use and care of animals were performed according to the Directive 2010/63/EU of the European Parliament and the German animal protection code. Permission was granted by local authorities (V 242 - 12956/2018).

Note that full information on the approval of the study protocol must also be provided in the manuscript.
